# Immunogenicity of rotavirus vaccine (Rotarix^TM^) in infants with environmental enteric dysfunction

**DOI:** 10.1371/journal.pone.0187761

**Published:** 2017-12-27

**Authors:** Innocent Mwape, Samuel Bosomprah, John Mwaba, Katayi Mwila-Kazimbaya, Natasha Makabilo Laban, Caroline Cleopatra Chisenga, Gibson Sijumbila, Michelo Simuyandi, Roma Chilengi

**Affiliations:** 1 Center for Infectious Disease Research in Zambia, Lusaka, Zambia; 2 Department of Physiological sciences,University of Zambia, Lusaka, Zambia; 3 Department of Biostatistics, School of Public Health, University of Ghana, Legon, Accra, Ghana; 4 University of North Carolina at Chapel Hill, Chapel Hill, North Carolina, United States of America; University of Liverpool, UNITED KINGDOM

## Abstract

**Introduction:**

Deployment of rotavirus vaccines has contributed to significant declines in diarrheal morbidity and mortality globally. Unfortunately, vaccine performance in low-middle income countries (LMICs) is generally lower than in developed countries. The cause for this has been associated with several host and maternal factors including poor water sanitation and hygiene (WASH) status, which are predominant in LMICs. More recently, environmental enteric dysfunction (EED) has specifically been hypothesized to contribute to poor vaccine uptake and response. The aim of this study was to examine the association between serological biomarkers of EED and seroconversion to rotavirus vaccine in Zambian infants.

**Methods:**

This was a retrospective cohort study of 142 infants who had been fully immunized with Rotarix™, and had known seroconversion status. Seroconversion was defined as 4-fold or more increase in rotavirus-specific IgA titres between pre-vaccination and one month post-dose two vaccination. We performed ELISA assays to assess soluble CD14 (sCD14), Endotoxin Core IgG Antibodies (EndoCAb), intestinal fatty acid binding protein (i-FABP) and Zonulin according to the manufacturers protocols. Generalised linear model with family-poisson, link-log and robust standard error was used to estimate the independent effects of biomarkers on seroconversion adjusting for important cofounders.

**Results:**

The median concentration of Zonulin, Soluble CD14, EndoCaB, and IFABP were 209.3 (IQR = 39.7, 395.1), 21.5 (IQR = 21.5, 21.5), 0.3 (IQR = 0.3, 0.3), and 107.7 (IQR = 6.4, 1141.4) respectively. In multivariable analyses adjusting for the independent effect of other biomarkers and confounders (i.e. age of child at vaccination, breast-milk anti-rotavirus IgA, infant serum anti-rotavirus IgG, and IgA seropositivity at baseline), there was strong evidence of about 24% increase in seroconversion due to doubling Zonulin concentration (Adjusted risk ratio (aRR) = 1.24; 95% CI = 1.12 to1.37; p<0.0001). Similarly, we found about 7% increase in seroconversion due to doubling IFABP concentration (aRR = 1.07; 95% CI = 1.02 to 1.13; p = 0.006).

**Conclusion:**

We found that high levels of zonulin and IFABP played a role in seroconversion. It is plausible that increased gut permeability in EED allows greater uptake of the live virus within the vaccine, but later consequences result in deleterious local structural distortions and malabsorption syndromes.

## Introduction

Diarrhea is the second largest killer of children in the world and rotavirus is the most common cause of severe diarrhea among children <5 years of age globally [1]. Rotavirus caused an estimated 233,000 deaths of children in 2013 alone, with the majority of these deaths occurring in low and middle-income countries (LMICs) [2]. Zambia records over 3,600 rotavirus-related deaths per year among children under 5 years [3].

Many LMICs are adding oral rotavirus vaccines (RVs) to their national immunization schedules to reduce the burden of rotavirus diarrhea [4]; however, RVs are proving to have lower immunogenicity, efficacy, effectiveness, and duration of protection in LMIC children [5]. For example, while in US children, RV effectiveness against hospitalization for rotavirus diarrhea was 87% (95% CI, 71%, 94%), in sub-Saharan Africa, clinical trials of the pentavalent (RV5) and monovalent (RV1) RVs showed efficacies were 39% (95% CI, 19%, 55%) and 61% (95% CI, 44%, 73%), respectively [6–13].

Several factors such as micronutrient deficiency, co-administration with oral poliovirus vaccines and maternal breast milk factors have been suggested as reasons for low immunogenicity of oral vaccines in developing countries [14, 15]. However, there is little known on the effect of intestinal mucosal integrity on seroconversion following administration of rotavirus vaccine [16].

Environmental enteric dysfunction (EED) is a syndrome of mucosal and sub-mucosal inflammation, reduced intestinal absorptive capacity and reduced barrier function, which is widespread in both adults and children residing in low and middle-income countries (LMICs) [17, 18]. Chronic inflammation due to EED has been associated with non-specific responses to oral vaccine antigens and result in clearance of the vaccine before sufficient induction of adaptive immunity [19–21]. Studies that have investigated EED have demonstrated intestinal architectural alterations such as crypt hyperplasia, blunting of the villi, and lymphocytic infiltration of the lamina propria [20, 22, 23]. Individuals with EED can often be asymptomatic of gastrointestinal diseases but may demonstrate underlying malabsorption and low grade inflammation that may potentially result in failure of oral vaccines [20, 24, 25].

Several biomarkers have been shown to be associated with, mucosal dysfunction of the small intestine in children under 5 years of age from developing countries [26]. Zonulin physiologically modulates tight junctions of enterocytes of the digestive tract [27]. This protein binds to a specific receptor on the surface of intestinal epithelia cells that induces tight junction disassembly and a subsequent increase in permeability of the intestinal epithelia [28]. Intestinal fatty acid binding protein (I-FABP) is a glycoprotein specifically secreted in circulation due to enterocyte damage [29]. Soluble CD14 (sCD14) is the glycoprotein expressed mainly on the surfaces of monocytes or macrophages which acts as a co-receptor along with Toll like receptor type 4 to which later binds lipopolysaccharides [30, 31]. Fourth, is Endotoxin Core IgG Antibodies (EndoCab); enhanced levels of this marker in serum may reflect a systemic inflammatory response [32].

We hypothesised that the levels of these intestinal inflammation biomarkers, measuring EED, may have an influence on seroconversion in infants receiving rotavirus vaccines. The objective of the study was to examine the association of Zonulin, I-FABP, sCD14 and EndoCab serum levels prior to vaccination with seroconversion in infants receiving rotavirus vaccine (Rotarix™ -GSK Biologicals, Belgium) in Lusaka, Zambia.

## Methods

### Study site and participants

In this study, serum samples collected from infants receiving rotavirus vaccination in a previously described study were used [5]. Briefly, the study was conducted at Kamwala clinic, a peri-urban health facility in Lusaka under which a prospective cohort of 420 infants aged between 6 to 12 weeks and receiving Rotarix™ vaccine was enrolled between April 2013 and March 2014. Blood was drawn from infants at baseline, before receiving the first dose of Rotarix™ and at one month post vaccine dose two at which seroconversion status was determined based on a four-fold or greater increase in rotavirus-specific IgA from baseline. Ethical approval was obtained from the University of Zambia Biomedical Research Ethics Committee as well as the Institutional Review Board of the University of North Carolina, at Chapel Hill USA. The study is registered at Clinical Trials.gov with NCT# 01886833.

**Fig 1:**
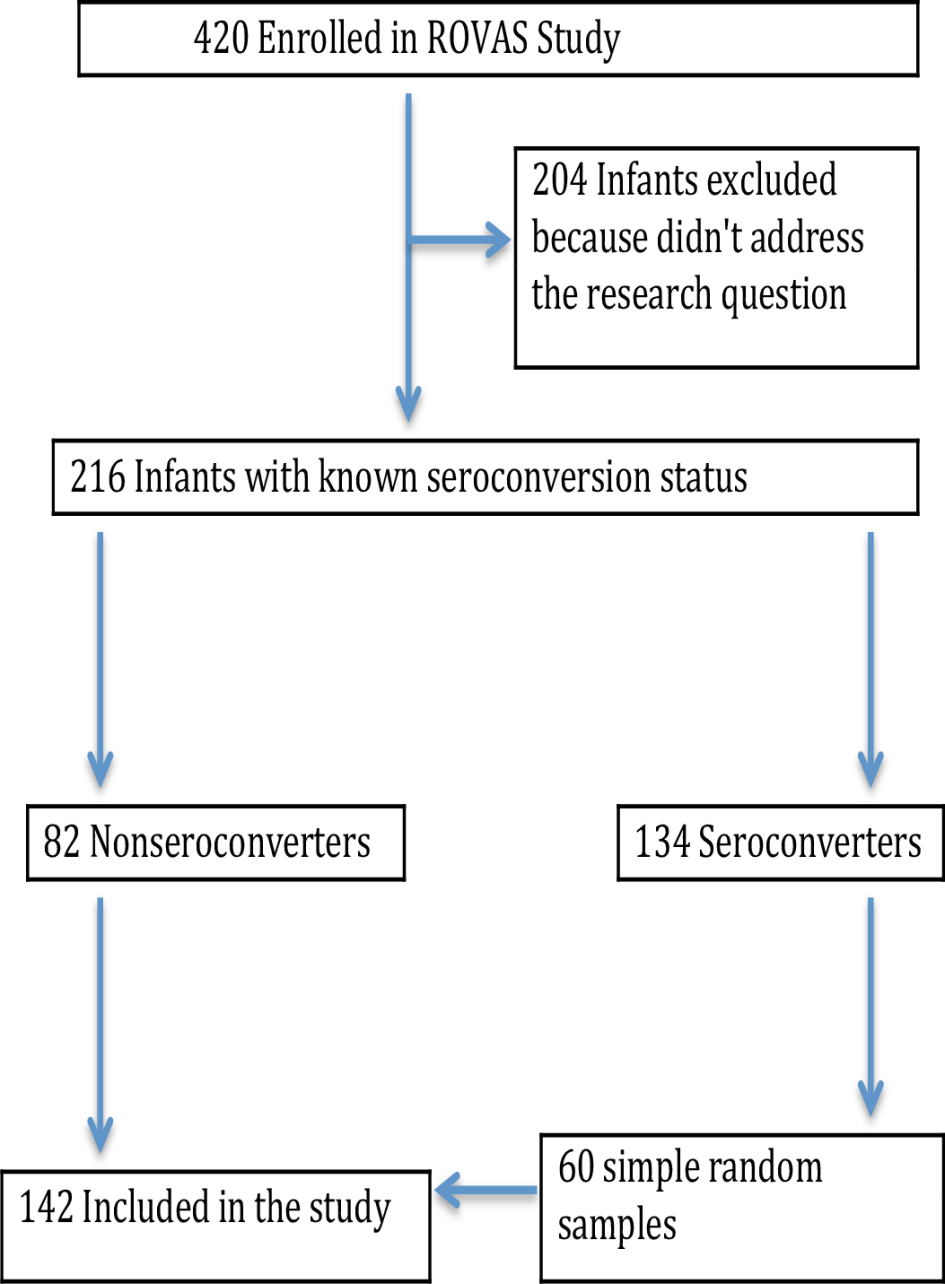
Infant recruitment and sample selection flow chart.

Baseline sera from infants with known seroconversion status were included and tested for presence of EED biomarkers. Of 420 enrolled infants, 216 had known seroconversion status: 82 classified as non-seroconverters, and 134 as seroconverters. Screening for EED biomarkers was done for all of the non-seroconverters and for 60 randomly selected seroconverters as illustrated in Fig 1.

### Post-hoc power calculation

With a seroconversion rate estimated at 60% in the general population [5], the study sample of 142 infants had 85% power to detect a reduction in seroconversion to 35% using a 2-sided Pearson Chi-Squared Test at 5% level of significance.

### Laboratory procedures

#### Measurement of serum IgA and IgG

Rotavirus-specific serum IgA and IgG were determined by an antibody capture ELISA assay as previously described [5]. Plates were coated with rabbit hyperimmune serum to rhesus rotavirus (RRV) and incubated with diluted RV1 strain or blotto (5% skim milk in phosphate-buffered saline [PBS]). Following incubaton, plates were washed and serially diluted serum samples in diluent buffer (1% skim milk and 0.5% [v/v] of 10% polyoxyethylene ether W1 in PBS) were added to the wells together with biotin-conjugated goat antihuman IgA antibodies. This was followed by the second incubation stage and washing afterwards. Extravidin was then added to all wells and incubated. After incubation, reactions were developed with 3,3′,5,5′-tetramethylbenzidine) and stopped with 1N hydrogen chloride. Optical density (OD) were read at 450 nm with an enzyme immunoassay reader. Calculation of IgA titres in serum were as the reciprocal of the highest dilution that gave a mean OD greater than the cut-off value (3 standard deviations above the mean OD of the negative control serum wells). RV-specific IgG in serum samples was tested and analysed in a similar way as IgA the only difference was that 0.5% normal rabbit serum was added to the biotin-conjugated goat antihuman IgG antibody solution.

#### Measurement of serological biomarkers of EED

Commercial enzyme linked immunosorbent assays (ELISA) kits were used to measure levels of sCD14, EndoCAb, I-FABP (Hycult Biotech, Uden, Netherlands) and Zonulin (Immundiagnostik AG, Bensheim Germany) according to the manufacturer’s instructions with modifications on sample dilutions. Undiluted plasma samples were used for measurement of sCD14 whereas samples were diluted 1:20 for Zonulin, 1:50 for Endocab and 1:10 for IFABP. Optical density was measured at 450nm (ELX808 BioTek) and concentrations were determined using assay standard curve.

### Statistical analysis

The primary outcome of interest was seroconversion defined as 4-fold or more increase in rotavirus-specific IgA titres between pre-vaccination and one month post-dose -2 vaccination. Rotavirus-specific IgA titres below limit of detection were imputed with 1 [5] before assessing the fold-increase. The exposures of interest were serological EED biomarkers of intestinal damage (IFABP), permeability (Zonulin) and microbial products of translocation (soluble CD14 and Endocab). We summarised the concentration of EED biomarkers using median and interquartile range. We used Fisher’s exact test to examine association of seroconversion with key infant and maternal factors. We used test for trend to assess dose-response relationship between the serological biomarkers of EED and seroconversion. The test for trend was performed by modelling the median concentration in each quartile of the biomarker concentration on seroconversion using a logit model.

Since this was a cohort study design we elected to estimate risk ratio using poisson with robust standard error. Therefore, Generalised linear model with family-poisson, link-log and robust standard error was used to estimate the independent effects of biomarkers on seroconversion adjusting for important cofounders. The biomarkers were modelled on log base 2 scale so that the effect would be doubling of the level of the biomarker. Values of biomarker concentrations below the limit of detection were imputed with half the lowest concentration for that biomarker before the log-transformation. The analysis was performed using Stata 15 (Statcorp, College Station, Texas, USA).

## Results

A total of 142 infant plasma samples were assessed for serological EED biomarkers. The median concentration of Zonulin, Soluble CD14, EndoCaB, and IFABP were 209.3 (IQR = 39.7, 395.1), 21.5 (IQR = 21.5, 21.5), 0.3 (IQR = 0.3, 0.3), and 107.7 (IQR = 6.4, 1141.4) respectively (Table 1).

**Table 1:** Seroconversion at post dose 2 by environmental enteric dysfunction status and key infant and maternal factors.

Biomarkers and Characteristics	Number of Infants (% of total)	No. (%) seroconverted	95% CI	Fisher's exact P-value
**Zonulin titre—Quartiles (median titre)**			** **	** **
Median (IQR)	209.3 (39.7, 395.1)			<0.0001*
1 (3.8)	36 (25)	6 (17)	(8, 33)	
2 (120.1)	35 (25)	16 (46)	(30, 62)	
3 (272.9)	35 (25)	21 (60)	(43, 75)	
4 (507.3)	35 (25)	23 (66)	(48, 80)	
**Soluble CD14 titre—Quartiles (median titre)**				
Median (IQR)	21.5 (21.5, 21.5)			0.189 *
1 (21.5)	130 (92)	63 (48)	(40, 57)	
2 (NIL) ^2^	NIL	NIL		
3 (NIL) ^2^	NIL	NIL		
4 (243)	11 (8)	3 (27)	(8, 60)	
**EndoCaB titre—Quartiles (median titre)**				
Median (IQR)	0.3 (0.3, 0.3)			**0.073** *
1 (0.3)	125 (89)	62 (50)	(41, 58)	
2 (NIL) ^2^	NIL	NIL		
3 (NIL) ^2^	NIL	NIL		
4 (13.3)	16 (11)	4 (25)	(9, 52)	
**IFABP titre—Quartiles (median titre)**				
Median (IQR)	107.7 (6.4, 1141.4)			**0.001** *
1 (6.4)	67 (47)	17 (25)	(16, 37)	
2 (15.6)	4 (3)	3 (75)	(18, 98)	
3 (534.3)	35 (25)	22 (63)	(46, 77)	
4 (2555.7)	35 (25)	24 (69)	(51, 82)	
**Age of child at vaccination (Weeks)**				
Median (IQR)	6 (6, 7)			**0.034**
<7	91 (65)	49 (54)	(43, 64)	
7+	50 (35)	17 (34)	(22, 48)	
**Sex of child**				
Female	66 (47)	28 (42)	(31, 55)	0.398
Male	75 (53)	38 (51)	(39, 62)	
**Infant serum anti-rotavirus IgG titre—Quartiles (median titre)**				
Median (IQR)	5120 (2560, 10240)			0.322
1 (2560)	49 (35)	27 (55)	(41, 69)	
2 (5120)	32 (23)	15 (47)	(30, 64)	
3&4 (10240) ^1^	59 (42)	24 (41)	(29, 54)	
**Seropositivity at baseline (IgA > = 1:40)**				
No	107 (76)	54 (50)	(41, 60)	**0.167**
Yes	34 (24)	12 (35)	(21, 53)	
**Age of mother (Years)**				
Median (IQR)	24 (22, 29)			0.590
16–19	18 (13)	7 (39)	(19, 63)	
20–24	56 (40)	28 (50)	(37, 63)	
25–29	33 (23)	13 (39)	(24, 57)	
30–39	34 (24)	18 (53)	(36, 69)	
**Maternal HIV Status**				
Negative	88 (62)	42 (48)	(37, 58)	0.862
Positive	53 (38)	24 (45)	(32, 59)	
**Breast-milk anti-rotavirus IgA—Quartiles (median titre)**				
Median (IQR)	160 (80, 320)			**0.053**
1 (80)	54 (40)	32 (59)	(46, 72)	
2 (160)	30 (22)	11 (37)	(21, 55)	
3 (320)	28 (21)	12 (43)	(26, 62)	
4 (640+)	24 (18)	7 (29)	(14, 50)	
**Total**	**141**	**66 (47)**	**(39, 55)**	** **

^1^ 3^rd^ and 4^th^ Quartiles were combined because the frequencies were small

^2^ Over 90% of titres were below limit of detection and therefore 25^th^, 50^th^, 75^th^ percentiles were the same as shown in the interquartile range (IQR)

* P-values were calculated using logit model where median titres were used to test for trend

In univariable analyses, there was strong evidence that high levels of Zonulin (test for trend p<0.0001) and IFABP (test for trend p = 0.001) were associated with increased probability of seroconversion, while there was no evidence at 5% level of significance that high levels of sCD14 (p = 0.189) and EndoCab (p = 0.073) were associated with seroconversion (Table 1, Table 2).

**Table 2:** Independent effects of markers of environmetal enteric dysfunction on seroconversion post dose 2 among rotavirus vaccinated infants aged 6–11 weeks.

Biomarkers	Crude RR (95%CI)	P-value	Adjusted RR (95%CI) ^1^	Adjusted P-value
**Zonulin**	** **	** **	** **	** **
Titre transformed to log base 2	1.26 (1.14, 1.40)	**<0.0001**	1.24 (1.12, 1.37)	**<0.0001**
**Soluble CD14**				
Titre transformed to log base 2	0.91 (0.71, 1.16)	0.438	0.91 (0.73, 1.13)	0.380
**EndoCaB**				
Titre transformed to log base 2	0.92 (0.79, 1.08)	0.307	0.98 (0.85, 1.14)	0.807
**IFABP**				
Titre transformed to log base 2	1.11 (1.06, 1.17)	**<0.0001**	1.07 (1.02, 1.13)	**0.006**

^1^ Estimates were adjusted for biomarkers (transformed on log base 2); and Age of child at vaccination (binary); Breast-milk anti-rotavirus IgA (transformed on log base 2); Seropositivity at baseline (IgA > = 1:40) (binary); Infant serum anti-rotavirus IgG (transformed on log base 2)

In multivariable analyses adjusting for the independent effect of other biomarkers and confounders (i.e. age of child at vaccination, breast-milk anti-rotavirus IgA, infant serum anti-rotavirus IgG, and IgA seropositivity at baseline), there was strong evidence of about 24% increased in seroconversion due to doubling Zonulin concentration (Adjusted risk ratio (aRR) = 1.24; 95% CI = 1.12 to1.37; p<0.0001) (Table 2). Similarly, we found about 7% increase in seroconversion due to doubling IFABP concentration (aRR = 1.07; 95% CI = 1.02 to 1.13; p = 0.006).

## Discussion

We report findings of the influence of intestinal inflammation biomarkers on seroconversion in infants receiving rotavirus vaccines. We found that high levels of Zonulin and IFABP were strongly associated with vaccine seroconversion. We also found very low levels of EndoCAb and sCD14, which were not associated with seroconversion.

Zonulin and IFABP biomarkers are indicators of the integrity of the “Gate” (intestinal barrier). Increased serum concentration reflects “Openness of the gate” and thus increased leaking from the intestinal lumen into lamina propria. We think this implied easy vaccine uptake and thus resulted into “better seroconversion”. Zonulin, as a tight junction modulator protein, is secreted due to antigen binding to receptor on intestinal epithelial cell surface, which later binds to EGF receptor (EGFR) via proteinase-activated receptor 2 (PAR_2_) [33]. Activation of the two receptors initiates the cascade reaction resulting in tight junction protein disengagement, this then enhances intercellular intestinal permeability [33, 34].

Elevated levels of zonulin in serum has been found to be associated with rotavirus infection in infants in Poland [35]. Further, it has been demonstrated that Dendritic Cells (DC) open the tight junctions between epithelial cells, send dendrites directly in the lumen for antigen sampling [36–38], suggesting that increased permeablility evidenced by high levels of Zonulin facilitates sending DC processes in lumen for antigen upatake. Also, antigen-nonspecific transport occurs through transcellular or paracellular pathways when the tight junction becomes more permeable or damaged by environmental factors. This increased uptake of antigens may occur as a result of allergic enteropathy, and other environmental factors that activate inflammatory cascades [39–41].

On the other hand, intestinal fatty-acid binding protein (I-FABP) is expressed in epithelial cells of the mucosal layer of the small intestine tissue [42]. When intestinal mucosal damage occurs due to presence of microbes or dietary antigens, gut barrier integrity can be disrupted leading to increased intestinal permeability [43]. This breach of intestinal barrier facilitates increased uptake of microbial products as well as live vaccines. These mechasims were supported by our findings that high levels of Zonulin and IFABP was associated with seroconversion. We think the high levels of Zonulin and IFABP implied early infant exposure to poor sanitation and hygiene, and therefore exposure to pathogens causing intestinal inflammation.

The timing of the samples just before the first vaccine dose was appropriate as it reflects the EED status of the internal milieu. We postulated that if the live oral vaccine is taken around such a time, the increased translocation of luminal microbes and their products actually facilitated better vaccine uptake. This thinking is consistent with the known mechanism for natural infections [17, 20, 21]. Our finding is consistent with that of a Bangladeshi study in which IFABP had positive association with oral chorela vaccine [44].

However, soluble CD14 and Endocab are markers of responses to lipopolysaccharides (LPS), a product of gram negative bacterial cell wall, which indicate “Gut leakage”. It has been postulated that high levels of sCD14 in the plasma are reflective of LPS exposure [45]. The low levels of soluble CD14 and Endocab found in our study may suggest that infants were less exposed to intestinal bacterial infection.

We acknowledge some limitations in our study. First, stool biomarkers were not used to asses EED [46]. Second, we did not assess the intestinal microbiota, which has been shown to have an effect on vaccine responses among infants [47]. Third, there may be other unmeasured confounders that could impact immune responses. Despite these limitations, we believe that the findings are relevant to warrant future studies establishing the levels of Zonulin and IFABP at which immune activation due to increased permeability does not lead to negative association of vaccine seroconversion.

## Conclusion

We found that high levels of zonulin and IFABP played a role in seroconversion. It is plausible that increased gut permeability in EED allows greater uptake of the live virus within the vaccine, but later consequences result in deleterious local structural distortions and malabsorption syndromes.

## Supporting information

S1 File (ZIP)

S2 File (ZIP)
