# Supplementary material for: Immunogenicity of rotavirus vaccine (RotarixTM) in infants with environmental enteric dysfunction
Source: PLoS One. 2017 Dec 27;12(12):e0187761. doi: 10.1371/journal.pone.0187761 (PMC5744930; doi:10.1371/journal.pone.0187761)
Supplement: S1 File — (ZIP) [file pone.0187761.s001.zip › SI1/30_03_2017_Immune response to rotavirus vaccine 12mo post vaccination in Zambian children_Final.docx]

Rotavirus immunity 12 months after vaccination in Zambian children.

Michelo Simuyandi^1^, Roma Chilengi^1, 2*^, Katayi M Kazimbaya^1^, Natasha Laban^1^, Caroline C Chisenga^1^, Baoming Jiang^3^, Samuel Bosomprah^1, 4^, Sylvia Becker-Dreps^2^.

1. Centre for Infectious Disease Research in Zambia, Lusaka, Zambia.
2. University of North Carolina at Chapel Hill, Chapel Hill, North Carolina, United States of America.
3. Centres for Disease Control and Prevention, Atlanta, Georgia, United States of America.
4. Department of Biostatistics, School of Public Health, University of Ghana, Legon, Accra

* [Roma.Chilengi@cidrz.or](mailto:Roma.Chilengi@cidrz.or)g

**Abstract**

Introduction

Oral rotavirus vaccines have shown low efficacy and effectiveness in low-middle income countries with protection waning in the first 2 years. There is limited research on the longevity of immunogenicity and factors associated with the sustained immunity in developing countries. Using a cohort of mother-infant pairs, we determined the longevity of rotavirus-specific IgA titres following immunization with the monovalent rotavirus vaccine (RV1) and factors associated with maintaining high anti-rotavirus IgA titres at 12 months of age.

Methods

One hundred and forty-four out of the 420 mother-infant pairs that were recruited when the infant was age 6–12 weeks in Lusaka were included in this study. We collected sociodemographic and clinical information, serum and maternal breastmilk samples at baseline, and additional serum samples one month post second dose of rotavirus vaccine (RV1) and at 12 months of age. Quarterly anthropometric data was also collected on infants from 14 weeks to 12 months of age. Determination of anti-rotavirus immunological titres of IgG and IgA in breast milk and infant serum was done using ELISA methods. Seroconversion was defined as a fourfold increase in anti-rotavirus IgA titre one month after the second dose of RV1 compared with the titre recorded at baseline. Protective immunity at 12 months of age was defined as >1:800 anti-rotavirus IgA titres at 12 months. We examined the associations between demographic, anthropometric, and baseline IgG and IgA anti-rotavirus titres in infant serum and breast milk and sustained immunogenicity at 12 months of age. Antibody variables were modelled on log-base 2. We used stepwise logistic regression with backward selection algorithm for the generalised linear model with log-link binomial-family to identify factors, which were independently associated with maintaining high antibody titres at 12 months of age.

Results

For each doubling of the baseline infant anti-rotavirus IgG there was an increased odds of protective immunity at 12 months (adjusted Odds Ratio [aOR] 1.72 [95% CI: 1.29, 2.29]. Stunting showed a strong positive association with protective immunity at 12 months (aOR 2.20 [95%CI: 0.97, 5.01]. A doubling of baseline infant anti rotavirus IgA titres showed a negative association (aOR 0.80 (95% CI: 0.64, 1.01) but did not attain statistical significance.

Conclusions

Children with higher baseline IgG titres and those who were stunted had increased odds of attaining protective immunity at 12 months.

Words: 380

Key words: protective immunity, immunogenicity, rotavirus vaccine, diarrhoea

**Introduction**

Globally, rotavirus is the most common cause of severe diarrhoea among children under the age of five [1]. In 2013 alone, it accounted for an estimated 215, 000 annual child deaths; with 56% of these in Sub-Saharan Africa [2]. While the primary burden of rotavirus disease occurs during the first year of life, a substantial burden of disease is experienced during the second year of life [1]**.** Rotavirus vaccine (RV) has been recommended for routine use in national expanded immunization programs (EPIs) worldwide [3]. Up to 86 countries are routinely using RV, with approximately 50% of African countries having introduced RV into their EPIs [4]. Since introduction of RV, there have been notable reductions in the number of deaths due to rotavirus diarrhoea in children under 5 years of age globally, from an estimated 528,000 (95% CI: 465,000–591,000) in 2000 to 215,000 (95% CI:,197,000–233,000) in 2013 [2]. In Zambia, the rotavirus prevalence among children presenting to the University Teaching Hospital with acute gastroenteritis declined from 40% in pre-vaccine years to 30% in 2013 and 25% in 2014 [5]. Despite this impact, RV efficacy has been shown to be consistently lower in low and middle income countries (LMIC) as compared to high income countries (HIC) (40-60% in LMIC vs. 80-90% in HICs) [6–8]. Research also shows marked differences in immunogenicity following rotavirus immunization [7,9–11]. Understanding the factors which lead to poor RV performance in LMIC may inform interventions that further reduce the burden of rotavirus disease.

Several factors have been postulated to be responsible in part, for the observed reduction in efficacy and effectiveness and varied immunogenicity of RV in developing countries. These factors include infant factors such as: (i) micronutrient or protein-energy malnutrition [12], (ii) effects of chronic environmental enteropathy [13], (iii) genetic factors (e.g. histo-blood group antigens) [14], (iv) concurrent infection with other enteric pathogens [15], (v) dysbiosis of the gut microbiome [16,17], (vi) co-administration with the oral polio vaccine [18]; and maternal factors, such as (i) interference by transplacental antibodies(IgG) [9], (ii) interference by breast milk antibodies and other non-immunological breastmilk components [19–21].

Clinical trials and case-control studies conducted in Malawi and Nicaragua show lower RV effectiveness in the second year of life as compared to the first year of life, while in the US, RV effectiveness is maintained in the second year of life [22–25]. Longitudinal studies of immunized children can provide additional information about long-term protection conferred by RV. One challenge to these studies which rely on immunological outcomes, is that there is not an established immunological correlate of protection against rotavirus infection or disease, although anti-rotavirus IgA is generally accepted as the best immunological marker available [26]. The classic cohort studies of rotavirus in Mexico children by Velazquez, et al, found that a geometric mean titer (GMT) >800 of anti-rotavirus IgA was associated with protection against subsequent severe rotavirus gastroenteritis [27].

The objective of this study was to report evidence of long-term immunogenicity after routine immunisation with monovalent RV (Rotarix®, GSK, Brentford, UK, RV1) in a Zambian population, and examine factors associated with maintaining high titres at 12 months of age. The understanding of the longevity of anti-rotavirus IgA titres at 12 months of age will also contribute to knowledge around the potential need for a third booster dose of rotavirus vaccine at 9 months of age in this population.

**Materials and methods**

**Study site and participants**

A previously described mother–infant pair population from a prospective cohort study [19] was used in this study. Healthy mother-infant pairs (irrespective of the mothers HIV status) were recruited as they presented to the health facility (Kamwala clinic) for routine immunisation service, with infants eligible to receive oral rotavirus vaccine at 6 weeks of age. The study was conducted between April 2013 and March 2015. Infants with known underlying immunosuppressive conditions and clinically determined to be failing to thrive were excluded. All vaccines received by infants in the study were provided as part of standard-of-care according to the expanded program on the immunization (EPI) schedule in Zambia. RV1 was administered at 6-8 and 14 weeks of age, concurrently with 13-valent pneumococcal conjugate vaccine (PCV-13).

The study protocol was approved by the University of Zambia Biomedical Research Ethics Committee, University of North Carolina at Chapel Hill Institutional Review Board and the Zambian Ministry of Health. The study was conducted in accordance with the principles of the Declaration of Helsinki and in compliance with good clinical practice guidelines; ClinicalTrials.gov registration number NCT 01886833.

**Clinic procedures and follow up**

Figure 1 provides a schema of study overview, which has already been reported (19). Briefly, mother-infant pairs were enrolled at a routine 6 week immunisation visit. At baseline, demographic information was collected, physical examination was performed, and serum samples were collected before infants received the first RV1 dose. At the same visit, mothers provided a sample of breast milk. A second RV1 dose was administered one month after immunization, and blood samples were collected from infants one month after the second dose and at 12 months of age. All specimens were collected and transported on ice and processed within 4 hours, before being frozen at -80°C until serological testing.Infants were followed up quarterly until after 24 months of age. At each of these follow-up visits, physical examination including assessment of anthropometric indices was done. Mothers were questioned about any diarrhoea episodes at each visit and were advised to bring the child to the facility in case of any illness.

**Fig 1. Shows clinical procedures and follow up activities over the study period.**

**Laboratory procedures**

Anti-rotavirus IgA in breast milk samples and anti-rotavirus serum IgA and IgG were determined by an antibody capture ELISA assay as described previously [19] at the Centre for Infectious Disease Research in Zambian (CIDRZ) laboratories. The reciprocal of the highest dilution that gave a mean OD greater than the cut-off value (3 standard deviations above the mean OD of the negative control serum wells) was used to calculate the immunoglobulin titres in breast milk and serum.

**Definition of terms**

“Diarrhoea” was defined as mother reporting that the child was passing stool more watery than usual, and at a frequency of >3 in a 24-hour period. At each visit, the mother was asked about diarrhoea in the period preceding her visit to clinic; or when she directly reported to the clinic because of an episode of diarrhoea.

“Seroconversion” was defined as a four fold or greater increase in anti-rotavirus IgA titers one month post the second RV1 dose compared to baseline titres.

“Protective immunity” is the primary outcome measure and was defined as infant serum anti-rotavirus IgA titre >1:800. This threshold was previously reported as a cut off at which child is 100% protected from moderate to severe rotavirus diarrhoea [27].

“Sustained immunogenicity” is the secondary outcomes measure, and was defined among those infants who seroconverted as the maintenance of the seroconversion definition at 12 months of age (at least a four-fold increase of anti-rotavirus IgA titres at 12 months as compared to baseline titres.).

“Seropositive” was defined as a baseline anti-rotavirus IgA titer >1:40.

“Stunting” was defined as Height-for-age z-score (HAZ) below - 2SD determined at 6 months infant age.

**Sample size calculations**

We calculated the post-hoc power for a given sample size of 144 available for this study. For a 40% prevalence of protective immunity at 12 months, this study has 80% power to detect an increase to 63% in the fraction of infants with protective immunity at 12 month using two-sided Pearson’s chi-squared test at 5% level of significance

**Statistical analysis**

We summarised the primary and secondary outcomes using proportions, which were compared between categories of key infant and maternal characteristics using Pearson’s chi-squared tests. We used simple logistic regression to test for trend; the breast milk anti-rotavirus IgA and baseline infant serum anti-rotavirus IgG titres were each modelled on a continuous scale using the median of the quartiles. We used stepwise with backward selection algorithm with generalised linear model (log-link and Poisson-family) with robust standard error to identify factors, which were independently associated with probability of sustained immunogenicity at 12 months of age; breast milk IgA and infant IgG were modelled on log base 2 scales so that the effect is a doubling of the titers of breast milk IgA and infant serum IgG. Variables were removed at p<0.2 using the likelihood ratio test. We also performed a sensitivity analysis in which we excluded infants who were seropositive at baseline. In an exploratory analysis, we used ANOVA F test to assess how protective immunity at 12 month varies with reported diarrhoea for each subgroup of post dose 2 seroconversion status. All analyses were performed using Stata 14 MP (StataCorp, College Station, Texas, USA).

**Results**

Out of a total of 420 mother-infant pairs who were enrolled in the study, 333 of the infants had baseline anti-rotavirus IgA measured and 236 of these infants had their anti-rotavirus IgA measured at 3 months (Fig. 2). A total of 144 had anti-rotavirus IgA determined at 12 months and were included in this analysis, as shown in fig 2. Of the 144 included in the study, 522% were female, the median age at vaccination was 6 weeks (IQR: 6, 7), 22% were seropositive at baseline, 62% seroconverted following receipt of the full course of RV1. Out of the 62% (88/143) who seroconverted following receipt of the full course of RV1, 41% (36/88) maintained sustained immunogenicity at 12 months of age.

**Fig 2. Study Participants Flow chart**

**Characteristics associated with protective immunity at 12 months** The bivariate analysis shows that higher baseline anti-rotavirus IgG titres and stunting were significantly associated with protective immunity at 12 months using the cut off of anti-rotavirus IgA titer >1:800 (*p*<0.001 and *p*=0.05 respectively, Table 1).

**Table 1: Protective immunity at 12 months by key infant and maternal factors**

| **Characteristics** | **Number of Infants (% of total)** | **Protective immunity^$^ (defined as IgA titer > 1:800) at month 12** | |
| --- | --- | --- | --- |
|  |  | Proportion (95%CI) | Chi2 P-value |
| **Age of child at vaccination (Weeks)** | | | |
| Median (IQR) | 6 (6, 7) | | 0.94 |
| <7 | 94 (66) | 44 (34, 54) |  |
| ≥7 | 49 (34) | 45 (31, 59) |  |
| **Sex of child** | | | |
| Female | 74 (52) | 46 (35, 57) | 0.71 |
| Male | 69 (48) | 43 (32, 55) |  |
| **Baseline infant serum anti-rotavirus IgG titre - Quartiles (median)** | | | |
| Median (IQR) | 5120 (2560, 10240) | | **<0.001*** |
| 1 (2560) | 49 (34) | 31 (19, 45) |  |
| 2 (5120) | 29 (20) | 41 (25, 60) |  |
| 3 (10240) | 36 (25) | 28 (15, 45) |  |
| 4 (20480) | 30 (21) | 90 (73, 97) |  |
| **Seropositivity at baseline (IgA >1:40)** | | | |
| No | 111 (78) | 47 (38, 56) | 0.35 |
| Yes | 32 (22) | 38 (22, 56) |  |
| **Seroconversion status after second vaccine dose** | | | |
| No | 55 (38) | 51 (38, 64) | 0.24 |
| Yes | 88 (62) | 41 (31, 52) |  |
| **Height-for-age below - 2SD at 6 month** | | | |
| Mean z score(SD) | -1.81 (1.40) | |  |
| No | 75 (59) | 38 (28, 50) | **0.05** |
| Yes | 52 (41) | 56 (42, 69) |  |
| **Age of mother (Years)** | | | |
| Median (IQR) | 25 (21, 29) | | 0.61 |
| 16-19 | 20 (14) | 50 (29, 71) |  |
| 20-24 | 49 (34) | 50 (36, 64) |  |
| 25-29 | 39 (27) | 41 (27, 57) |  |
| 30-39 | 35 (24) | 37 (23, 54) |  |
| **Maternal HIV Status** | | | |
| Negative | 90 (63) | 44 (34, 54) | 0.88 |
| Positive | 53 (37) | 45 (32, 59) |  |
| **Maternal CD4 count if HIV+** | | | |
| Median (IQR) | 488 (396, 582) | |  |
| 350+ | 28 (80) | 50 (31, 67) | 0.31 |
| <350 | 7 (20) | 29 (6, 72) |  |
| **Breast milk anti-rotavirus IgA - Quartiles (median)** | | | |
| Median (IQR) | 160 (80, 320) | |  |
| 1 (80) | 53 (38) | 40 (27, 53) | 0.24* |
| 2 (160) | 34 (24) | 56 (39, 72) |  |
| 3 (320) | 28 (20) | 54 (35, 71) |  |
| 4 (640+) | 25 (18) | 32 (17, 53) |  |
| **Reported diarrhoea** | | | |
| 0 | 74 (52) | 43 (32, 55) | 0.27 |
| 1 | 65 (45) | 43 (31, 55) |  |
| 2+ | 5 (3) | 80 (25, 98) |  |
| **Total** | **144 (100)** | **44 (36, 53)** |  |
| * Simple logistic regression was used to test for trend; the breastmilk IgA and infant RV IgG were each modelled on a continuous scale using median of the quartiles  ^$^Protective immunity” is the primary outcome measure and was defined as infant serum anti-rotavirus IgA titre >1:800 | | | |

**Factors associated with sustained immunogenicity**

The proportion of males (91%) who has sustained immunogenicity at 12 months was higher than females (80 %) even though the difference was not significant. There was a high proportion of children with sustaining immunogenicity among those classified as stunted at 6 months of age(96%) than those who were no stunted(88%). Children with Maternal HIV status that was positive had higher proportion of sustained immunogenicity compared to their counterparts from mothers who were HIV negative but the difference was not significant. Having a maternal CD4+ count of ≥350 had higher proportion of sustained immunogenicity (100%) compared to those with those with CD4+ counts of <350 (75%) (P=0.06)

**Table 2. Percentage of children that sustained immunogenicity at 12 months among those who seroconverted after the second rotavirus vaccine by key infant and maternal factors, Lusaka.**

| **Characteristics** | **Number of Infants that seroconverted after second dose** | **Children with sustained immunogenicity at 12 months** | | |
| --- | --- | --- | --- | --- |
|  |  | Number (%) | 95%CI | Chi2 P-value |
| **Age of child at vaccination (Weeks)** | | | | |
| Median (IQR) | 6 (6, 7) | | | |
| <7 | 62 | 53 (85) | [74, 92] | 0.92 |
| 7+ | 26 | 22 (85) | [65, 94] |  |
| **Sex of child** | | |  |  |
| Female | 45 | 36 (80) | [65, 89] | 0.16 |
| Male | 43 | 39 (91) | [77, 97] |  |
| **Infant serum anti-rotavirus IgG titre - Quartiles (median)** | | | | |
| Median (IQR) | 5120 (2560, 10240) | | | |
| 1 (2560) | 37 | 31 (84) | [68, 93] | 0.34 |
| 2 (5120) | 17 | 13 (76) | [50, 91] |  |
| 3 (10240) | 21 | 18 (86) | [63, 96] |  |
| 4 (20480) | 13 | 13 (100) | * |  |
| **Height-for-age below - 2SD at 6 month** | | | | |
| Mean z score (SD) | -1.81 (-2.58, -0.88) | | | |
| No | 50 | 44 (88) | [75, 95] | 0.24 |
| Yes | 26 | 25 (96) | [76, 99] |  |
| **Age of mother (Years)** | | | | |
| Median (IQR) | 25 (21, 29) | | | |
| 16-19 | 14 | 11 (79) | [73, 94] | 0.74 |
| 20-24 | 31 | 28 (90) | [73, 97] |  |
| 25-29 | 20 | 17 (85) | [61, 95] |  |
| 30-39 | 23 | 19 (83) | [61, 94] |  |
| **Maternal HIV Status** | | | | |
| Negative | 60 | 49 (82) | [70, 90] | 0.17 |
| Positive | 28 | 26 (93) | [75, 98] |  |
| **Maternal CD4 count if HIV+** | | | | |
| Median (IQR) | 488 (396, 582) | | | |
| 350+ | 13 | 13 (100) | * | 0.06 |
| <350 | 4 | 3 (75) | [15, 98] |  |
| **Total** | **88** | **75 (85)** | **[76, 91]** |  |
| * Not possible to compute CI because all infants born to mothers in that category had maintained their status | | | | |

**Infant and maternal factors independently associated with protective immunity**

Using the multivariable analysis, we found that, doubling the level of baseline infant anti-rotavirus IgG was independently associated with 33% increase in probability of protective immunity at 12 months (with an adjusted risk ratio (aRR of 1.33; 95% CI: 1.13, 1.56; p-value<0.0001), while a doubling of baseline infant breastmilk anti-rotavirus IgA was associated with 10% reduction in probability of protective immunity at 12 months (aRR=0.90 (95% CI: 0.82, 0.99; p-value=0.04) (Table 3). There was borderline evidence of stunting being associated with 40% increase in probability of sustained immunogenicity at 12 months (aRR=1.40 (95%CI: 0.98, 2.01; p-value=0.07). In a sensitivity analysis, the associations between protective immunity and either baseline IgG remain similar, while the association between stunting remain disappears after excluding infants who were seropositive at baseline from the analysis (Table 3)

**Table 3: Effects of infant and maternal factors independent associated with probability of protective immunity at 12 months**

| **Factors** | **Protective immunity (defined as IgA titer > 1:800) at month 12** | | ***Protective immunity (defined as IgA titer > 1:800) at month 12 ^2^** | |
| --- | --- | --- | --- | --- |
|  | Adjusted risk ratio (95%CI) | P-value | Adjusted risk ratio (95%CI) | P-value |
| **Breast milk anti-rotavirus IgA** | | | | |
| Transformed to log base 2 ^1^ | 0.90 (0.82, 0.99) | 0.04 | 0.90 (0.80, 1.00) | 0.06 |
| **Infant serum anti-rotavirus IgG titre** | | | | |
| Transformed to log base 2 ^1^ | 1.33 (1.13, 1.56) | **<0.0001** | 1.37 (1.11, 1.68) | **<0.01** |
| **Height-for-age below - 2SD at 6 month** | | | | |
| No | reference | 0.07 | reference | 0.14 |
| Yes | 1.40 (0.98, 2.01) |  | 1.34 (0.91, 1.97) |  |
| ^1^ Effect corresponds with doubling the level of titre  ^2^ Infants who were seropositive at baseline were excluded from the analysis  * Sensitivity analysis | | | | |

**Reported diarrhoea and anti-rotavirus immunity at 12 months**

In our sample, children with 2 or more episodes of all-cause diarrhoea had higher anti-rotavirus IgA titres at 12 months of age. Geometric mean titre (GMT) (Fig 3 A) or proportions(Fig 3 B) with protective immunity at 12 months appears to be higher among infants who seroconverted and had reported two or more episodes of diarrhoea. However, the difference is not statistically significant by either (ANOVA p-value=0.28) or protective immunity (ANOVA p-value=0.25). Also, there was no evidence that the effect varies by seroconversion status either (GMT ANOVA interaction p-value=0.82; protective immunity ANOVA interaction p-value=0.58).

**Fig 3 shows: Rotavirus IgA immunogenicity and reported diarrhoea episodes. [A] infant serum IgA titre at 12 months by number of reported diarrhoea incidences per child; [B] Proportion of children with serum IgA titres >1:800 at 12 months**

There was no difference in geometric mean IgA titres at 12 months of age between children who were seropositive and those who were seronegative at baseline (Fig 4(A)) and no difference in geometric mean IgA titres at 12 months of age between those who had seroconverted at post dose 2 verses those who did not seroconvert (Fig 4 (B)).

**Fig 4: Geometric mean IgA titre (95% confidence interval) over one year period by sero status at baseline[A] and Geometric mean IgA titre (95% confidence interval) over one year period by post dose 2 seroconversion status[B].**

**Discussion**

Our study found that for each doubling of the baseline infant anti-rotavirus IgG there was an increased odds of protective immunity (anti-rotavirus IgA titre >800) at 12 months (adjusted Odds Ratio [aOR] 1.72 [95% CI: 1.29, 2.29]. Also, stunting showed a strong positive association (aOR 2.20 [95%CI: 0.97, 5.01] with protective immunity at 12 months. A doubling of baseline infant anti-rotavirus IgA titres showed a negative association (aOR 0.80 (95% CI: 0.64, 1.01).

The observed association between high infant IgG titres at baseline with sustained immunogenicity at 12 months, and between children who were stunted at 6 months of age with sustained immunogenicity at 12 months in this population was unexpected, but may be explained as follows. It is possible that infants who acquired higher levels of transplacental antibodies (IgG) against rotavirus did not respond well to RV, resulting in increased susceptibility to natural infection with rotavirus later during infancy, which resulted in higher anti-rotavirus IgA titres. This is consistent with previous reports [9,28,29] which have shown that transplacentally acquired IgG interferes with RV1 uptake. Our prior study did show an association between high baseline IgG titers and failure to seroconvert to RV1.

While the numbers were few, we children who had not seroconverted had higher IgA and proportions that attained protective immunity at 12 months compared to non-seroconverters even though the difference was not significant. This observation seems to be consistent with the protective effect of RV1 monovalent rotavirus vaccination against diarrhoea (in children who took up the vaccine). The observed high anti-rotavirus IgA titre at 12 months in children with more episodes of diarrhoea is also consistent with expected increase in titres as result of repeated RV1 rotavirus infection.

Children who were stunted (HAZ Z-score <-2SD) at 6 months had higher IgA titres at 12 months of age compared to children with moderate or no stunting. Stunting is an indicator of malnutrition and has been linked to poor immune status and being more prone to infections [30–32].

Maternal HIV status seems to play a role in rotavirus vaccine immunogenicity of children, a observed a higher proportion of children with sustained immunogenicity whose maternal HIV status was positive compared to those with a maternal HIV status that was negative. When this was stratified by CD4+ count, those with CD4+ counts ≥350 had higher proportion of maintaining immunogenicity compared to those with CD4+ counts of <350. This warrants further study since evidence is now emerging to suggested that while HIV exposed infants seems to have similar immune status and responses to the HIV unexposed during their early infant lives[10], there is a difference in risk mortality rates as they grow(ref).

Our results and interpretation are limited because of several factors. Most notably, we primarily followed immunological outcomes, since we did not determine etiology of the diarrhoea episodes. Further, we acknowledge that our use of reported diarrhoea as an outcome measure is subject to courtesy bias and recall bias[33,34] which could have led to either under or over reporting of incidence. (Therefore, due to our dependence on immunological outcomes, we could not appropriately compare rotavirus gastroenteritis episodes between groups. Finally, we experienced a high loss to follow up in the sample, due to the long period required to ascertain the final measurement.

Despite the limitations, we remain confident with our findings because our study was sufficiently powered to explore our hypothesis, we do not think that few numbers included in this study compared to those enrolled could affect our results and interpretation due to the fact that the proportion of characteristics (i.e seroconverted, seropositive) are similar and that we also have other collaborative evidence both within and outside the study supporting main findings in the study.

These results nonetheless, warrant more research in understanding the kinetics of antibody acquisition in children vaccinated with rotavirus vaccine in developing countries in order to determine optimal vaccination time points. Furthermore, determining the functionality (neutralisation and inhibition ability) of rotavirus specific IgA and IgG in infants over time during the first 12 months of life will help in determining the potential booster benefit of a third RV dose dose earlier than 12 months to protect children who may have not taken up the vaccine at earlier vaccination time points.

**Conclusions**

Within the limitations of the study, we found that children with higher baseline IgG titres and those who were stunted had increased odds of attaining sustained immunogenicity at 12 months.

**Acknowledgements**

We are grateful to the parents of all the infants who have participated in this study. The study team including Marcellina Hamikondo, Fridah Madhabi, Margaret Chisambi, Catherin Phiri and Annie Chikombo are acknowledged for their contribution to make this study possible. We also would like to thank Dr Baoming Jiang for the donation of the MA 104 cell line and Rabbit hyperimmune serum to rhesus rotavirus (RRV). The finding and conclusions in this report are those of the authors and do not necessarily represent the official positions of Centers for Disease Control and Prevention.

**Funding**

This study is funded by the National Institutes for Health (NIH), USA, through an R01 grant 1R01AI099601. Co-author SBD was supported by 1R56A1108515-01 from the NIH. The funders had no role in study design, data collection and analysis, decision to publish, or preparation of the manuscript.

**Author Contributions**

Conceived and designed the experiments: MS RC KM CCC.

Performed the experiments: MS KMK CCC NL

Analysed the data: MS RC SB SBD KMK CCC NL BJ

Contributed reagents/materials/analysis tools: RC MS BJ

Wrote the paper: MS RC SB SBD KMK CCC NL BJ

Provided reagents for laboratory tests: BJ NIH RO1 grant

**References**

1. Liu J, Platts-Mills JA, Juma J, Kabir F, Nkeze J, Okoi C, et al. Use of quantitative molecular diagnostic methods to identify causes of diarrhoea in children: a reanalysis of the GEMS case-control study. Lancet. Elsevier Ltd; 2016;388: 1291–1301. doi:10.1016/S0140-6736(16)31529-X

2. Tate JE, Burton AH, Boschi-Pinto C, Parashar UD, Agocs M, Serhan F, et al. Global, Regional, and National Estimates of Rotavirus Mortality in Children <5 Years of Age, 2000-2013. Clin Infect Dis. 2016;62: S96–S105. doi:10.1093/cid/civ1013

3. World Health Organization. Rotavirus vaccines WHO position paper: January 2013 - Recommendations. Wkly Epidemiol Rec. 2013;31: 6170–6171. doi:10.1016/j.vaccine.2013.05.037

4. World Health Organization (WHO). Vaccine Introduction Status. In: www.who.int/immunization/monitoring_surveillance/VaccineIntroStatus.pptx [Internet]. 2017 [cited 16 Feb 2017]. Available: www.who.int/immunization/monitoring_surveillance/VaccineIntroStatus.pptx

5. Mpabalwani EM, Simwaka CJ, Mwenda JM, Mubanga CP, Monze M, Matapo B, et al. Impact of Rotavirus Vaccination on Diarrheal Hospitalizations in Children Aged <5 Years in Lusaka, Zambia. Clin Infect Dis. 2016;62: S183–S187. doi:10.1093/cid/civ1027

6. Patel M, Shane AL, Parashar UD, Jiang B, Gentsch JR, Glass RI. Oral Rotavirus Vaccines: How Well Will They Work Where They Are Needed Most? J Infect Dis. 2009;200: S39–S48. doi:10.1086/605035

7. Madhi SA, Cunliffe NA, Steele D, Witte D, Kirsten M, Louw C, et al. Effect of Human Rotavirus Vaccine on Severe Diarrhea in African Infants. N Engl J Med. 2010;362: 289–298. doi:10.1056/NEJMoa0810625)

8. Beres LK, Tate JE, Njobvu L, Chibwe B, Rudd C, Guffey MB, et al. A Preliminary Assessment of Rotavirus Vaccine Effectiveness in Zambia. Clin Infect Dis. 2016;62: S175–S182. doi:10.1093/cid/civ1206

9. Moon SS, Groome MJ, Velasquez DE, Parashar UD, Jones S, Koen A, et al. Prevaccination Rotavirus Serum IgG and IgA Are Associated with Lower Immunogenicity of Live, Oral Human Rotavirus Vaccine in South African Infants. Clin Infect Dis. 2016;62: 157–165. doi:10.1093/cid/civ828

10. Groome MJ, Zell ER, Solomon F, Nzenze S, Parashar UD, Izu A, et al. Temporal Association of Rotavirus Vaccine Introduction and Reduction in All-Cause Childhood Diarrheal Hospitalizations in South Africa. Clin Infect Dis. 2016;62: S188–S195. doi:10.1093/cid/civ1204

11. Cheuvart B, Neuzil KM, Steele AD, Cunliffe N, Madhi A, Karkada N, et al. Association of serum anti-rotavirus immunoglobulin A antibody seropositivity and protection against severe rotavirus gastroenteritis Association of serum anti-rotavirus immunoglobulin A antibody seropositivity and protection against severe rotavirus gastr. 2014;5515. doi:10.4161/hv.27097

12. Perez-Schael I, Salinas B, Tomat M, Linhares AC, Guerrero ML, Ruiz-Palacios GM, et al. Efficacy of the human rotavirus vaccine RIX4414 in malnourished children. J Infect Dis. 2007;196: 537–540. doi:10.1086/519687

13. Gilmartin AA, Petri WAJ. Exploring the role of environmental enteropathy in malnutrition, infant development and oral vaccine response. Philos Trans R Soc Lond B Biol Sci. 2015;370. doi:10.1098/rstb.2014.0143

14. Nordgren J, Sharma S, Bucardo F, Nasir W, Günaydin G, Ouermi D, et al. Both lewis and secretor status mediate susceptibility to rotavirus infections in a rotavirus genotype-dependent manner. Clin Infect Dis. 2014;59: 1567–1573. doi:10.1093/cid/ciu633

15. Naylor C, Lu M, Haque R, Mondal D, Buonomo E, Nayak U, et al. Environmental Enteropathy, Oral Vaccine Failure and Growth Faltering in Infants in Bangladesh. EBioMedicine. 2015;2. doi:10.1016/j.ebiom.2015.09.036

16. Yu J, Ordiz MI, Stauber J, Shaikh N, Trehan I, Barnell E, et al. Environmental enteric dysfunction includes a broad spectrum of inflammatory responses and epithelial repair processes. C Cell Mol Gastroenterol Hepatol. Elsevier Inc; 2016;2: 158–174. doi:10.1016/j.jcmgh.2015.12.002

17. Blanton L V., Charbonneau MR, Salih T, Barratt MJ, Venkatesh S, Ilkaveya O, et al. Gut bacteria that prevent growth impairments transmitted by microbiota from malnourished children. Science (80- ). 2016;351. doi:10.1126/science.aad3311

18. Emperador DM, Velasquez DE, Estivariz CF, Lopman B, Jiang B, Parashar U, et al. Interference of Monovalent, Bivalent, and Trivalent Oral Poliovirus Vaccines on Monovalent Rotavirus Vaccine Immunogenicity in Rural Bangladesh. Clin Infect Dis. 2016;62: 150–156. doi:10.1093/cid/civ807

19. Chilengi R, Simuyandi M, Beach L, Mwila K, Becker-Dreps S, Emperador DM, et al. Association of Maternal Immunity with Rotavirus Vaccine Immunogenicity in Zambian Infants. PLoS One. 2016;11: e0150100. doi:10.1371/journal.pone.0150100

20. Moon S-S, Tate JE, Ray P, Dennehy PH, Archary D, Coutsoudis A, et al. Differential Profiles and Inhibitory Effect on Rotavirus Vaccines of Nonantibody Components in Breast Milk From Mothers in Developing and Developed Countries. Pediatr Infect Dis. 2013;32: 863–870. doi:doi:10.1097/INF.0b013e318290646d

21. Mwila K, Chilengi R, Simuyandi M, Permar SR, Becker-Dreps S. Contribution of maternal immunity to decreased rotavirus vaccine performance in low and middle income countries. Clin Vaccine Immunol. 2016; CVI.00405-16. doi:10.1128/CVI.00405-16

22. Staat MA, Payne DC, Donauer S, Weinberg GA, Edwards KM, Szilagyi PG, et al. Effectiveness of Pentavalent Rotavirus Vaccine Against Severe Disease. Pediatrics. 2011;128: e267–e275. doi:10.1542/peds.2010-3722

23. Bar-Zeev N, Jere KC, Bennett A, Pollock L, Tate JE, Nakagomi O, et al. Population Impact and Effectiveness of Monovalent Rotavirus Vaccination in Urban Malawian Children 3 Years after Vaccine Introduction: Ecological and Case-Control Analyses. Clin Infect Dis. 2016;62: S213–S219. doi:10.1093/cid/civ1183

24. Cunliffe NA, Witte D, Ngwira BM, Todd S, Bostock NJ, Turner AM, et al. Efficacy of human rotavirus vaccine against severe gastroenteritis in Malawian children in the first two years of life: A randomized, double-blind, placebo controlled trial. Vaccine. 2012;30: 36–43. doi:10.1016/j.vaccine.2011.09.120

25. Patel M, Pedreira C, De Oliveira LH, Umana J, Tate J, Lopman B, et al. Duration of Protection of Pentavalent Rotavirus Vaccination in Nicaragua. Pediatrics. 2012;130: e365–e372. doi:10.1542/peds.2011-3478

26. Jiang B, Gentsch JR, Glass RI. The role of serum antibodies in the protection against rotavirus disease: an overview. Clin Infect Dis. 2002;34: 1351–1361. doi:10.1086/340103

27. Velazquez RF, Matson DO, Guerrero ML, Shults J, Calva JJ, Morrow AL, et al. Serum Antibody as a Marker of Protection against Natural Rotavirus Infection and Disease. J Infect Dis. 2000;182: 1602–9.

28. Becker-dreps S, Vilchez S, Velasquez D, Hudgens MG, Zambrana LE. Rotavirus-Specific IgG Antibodies from Mothers’ Serum may Inhibit Infant Immune Responses to the Pentavalent Rotavirus Vaccine. Pediatr Infect Dis J. 2015;34: 115–116. doi:10.1097/INF.0000000000000481.Rotavirus-Specific

29. Appaiahgari MB, Glass R, Singh S, Taneja S, Rongsen-Chandola T, Bhandari N, et al. Transplacental rotavirus IgG interferes with immune response to live oral rotavirus vaccine ORV-116E in Indian infants. Vaccine. Elsevier Ltd; 2014;32: 651–656. doi:10.1016/j.vaccine.2013.12.017

30. Guerrant RL, Deboer MD, Moore SR, Scharf RJ, Aldo a M. The impovershied gut- a tripple burgen of diarrhoea, stunting and Chronic Disease. 2013;10: 220–229. doi:10.1038/nrgastro.2012.239.The

31. Prendergast AJ, Rukobo S, Chasekwa B, Mutasa K, Ntozini R, Mbuya MNN, et al. Stunting is characterized by chronic inflammation in zimbabwean infants. PLoS One. 2014;9. doi:10.1371/journal.pone.0086928

32. Keusch GT, Denno DM, Black RE, Duggan C, Guerrant RL, Lavery J V., et al. Environmental enteric dysfunction: Pathogenesis, diagnosis, and clinical consequences. Clin Infect Dis. 2014;59: S207–S212. doi:10.1093/cid/ciu485

33. Hassan E. Recall bias can be a threat to retrospective and prospective research designs. internet J Epidemiol. 2005;3: 1–11. doi:10.5580/2732

34. Arnold BF, Galiani S, Ram PK, Hubbard AE, Briceño B, Gertler PJ, et al. Optimal recall period for caregiver-reported illness in risk factor and intervention studies: A multicountry study. Am J Epidemiol. 2013;177: 361–370. doi:10.1093/aje/kws281
