# Supplementary figures and images for: Immunogenicity of rotavirus vaccine (RotarixTM) in infants with environmental enteric dysfunction
Source: PLoS One. 2017 Dec 27;12(12):e0187761. doi: 10.1371/journal.pone.0187761 (PMC5744930; doi:10.1371/journal.pone.0187761)

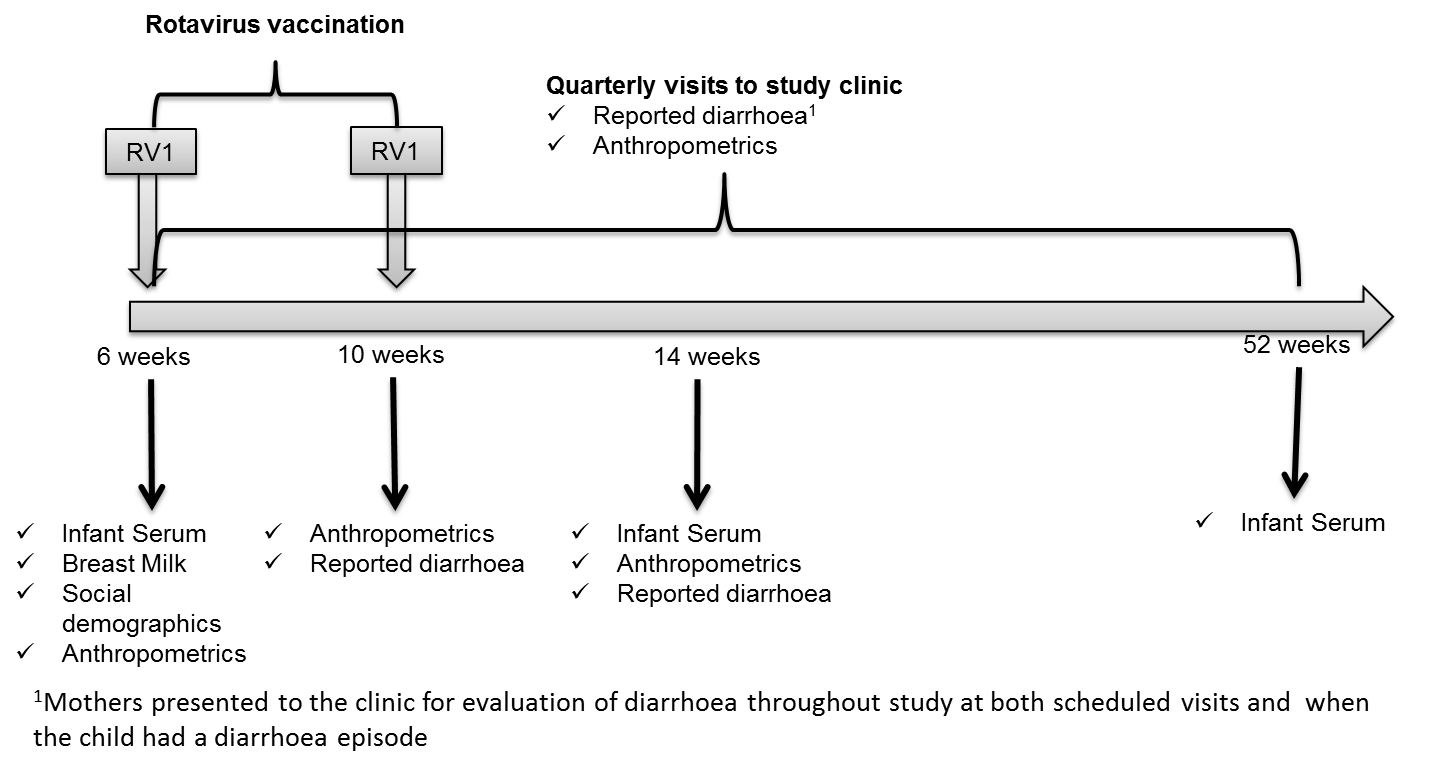

Supplement: S1 File — (ZIP) [file pone.0187761.s001.zip › SI1/Figure 1..tif]

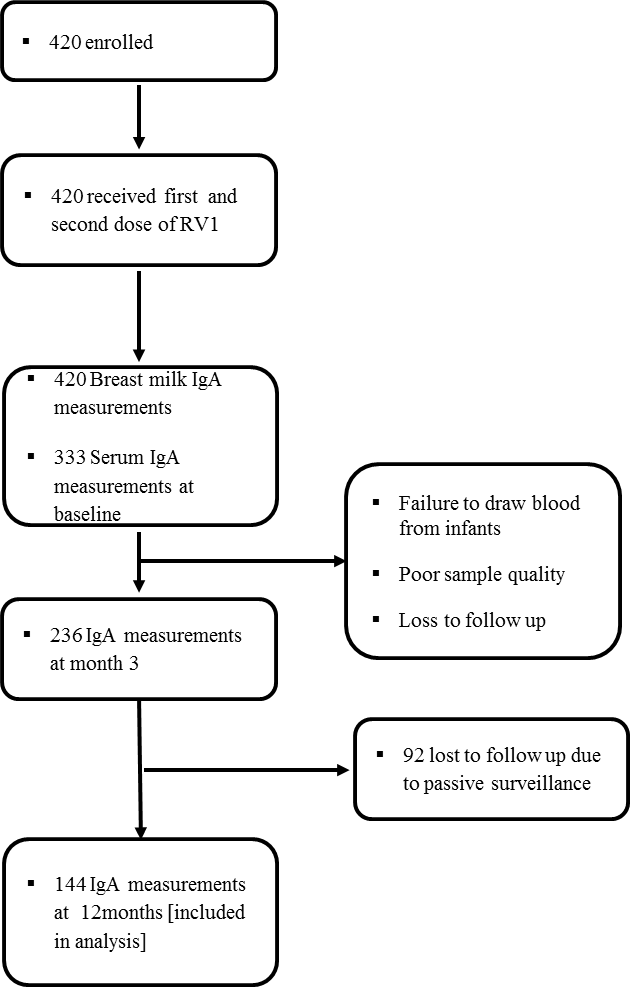

Supplement: S1 File — (ZIP) [file pone.0187761.s001.zip › SI1/Figure 2..tif]

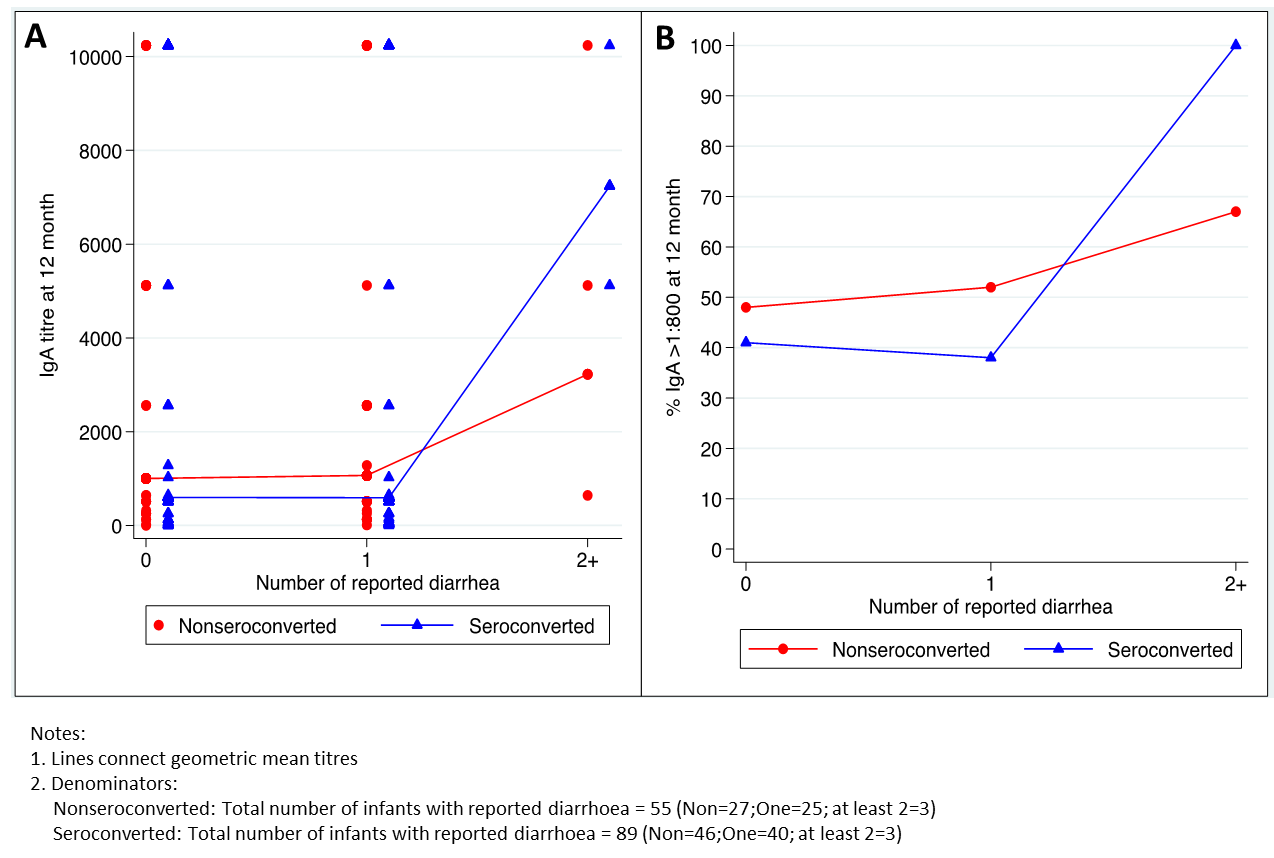

Supplement: S1 File — (ZIP) [file pone.0187761.s001.zip › SI1/Figure 3..tif]

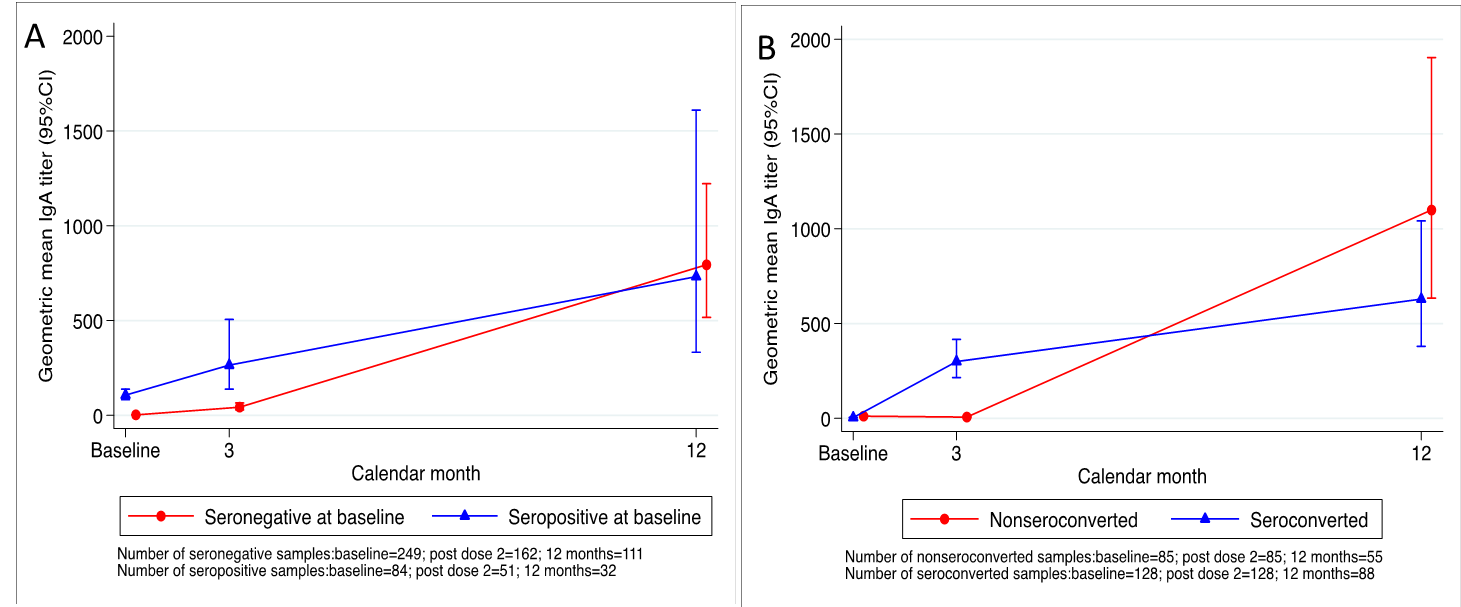

Supplement: S1 File — (ZIP) [file pone.0187761.s001.zip › SI1/figure 4.tif]

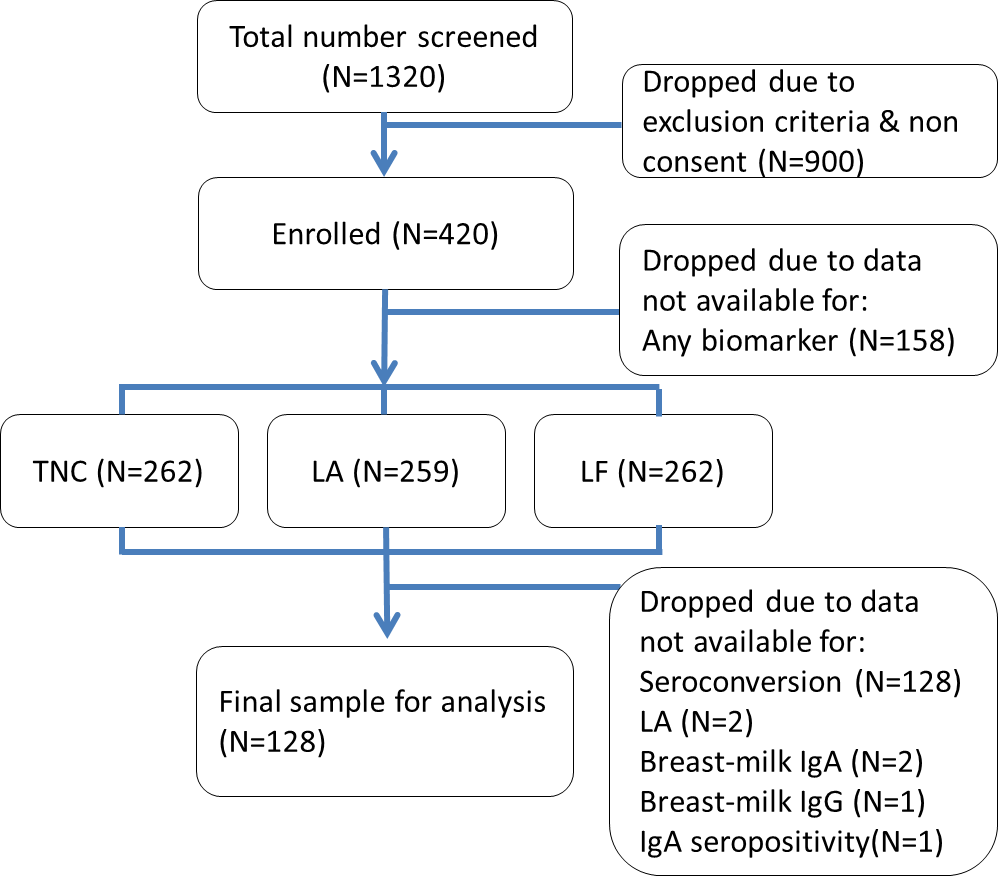

Supplement: S2 File — (ZIP) [file pone.0187761.s002.zip › Figure 1..tif]
