# Supplementary material for: Immunogenicity of rotavirus vaccine (RotarixTM) in infants with environmental enteric dysfunction
Source: PLoS One. 2017 Dec 27;12(12):e0187761. doi: 10.1371/journal.pone.0187761 (PMC5744930; doi:10.1371/journal.pone.0187761)
Supplement: S2 File — (ZIP) [file pone.0187761.s002.zip › Effect of non-immunological components .docx]

Effect of innate antiviral glycoproteins in breast milk on seroconversion to rotavirus vaccine (Rotarix) in children in Lusaka, Zambia

Katayi Mwila-Kazimbaya^1,2^, Miguel P. Garcia^1^, Natasha M. Laban^1^, Caroline C. Chisenga^1^, Sallie R. Permar^3^, Samuel Bosomprah^1,4^, Michelo Simuyandi^1^, Sody Munsaka^2^_,_ Roma Chilengi^1,5*^

^1^Center for Infectious Disease Research in Zambia, Lusaka, Zambia

^2^ Department of biomedical sciences, School of Health sciences, University of Zambia, Lusaka, Zambia

^3^Department of Paediatrics, Human Vaccine Institute, Duke University, Durham, North Carolina

^4^ Department of Biostatistics, School of Public Health, University of Ghana, Legon, Accra

^5^ University of North Carolina at Chapel Hill, School of Medicine, North Carolina, United States of America

*Corresponding author

E-mail: [Roma.Chilengi@cidrz.org](mailto:Roma.Chilengi@cidrz.org) (RC)

# Abstract

## Introduction

Rotavirus vaccines have been introduced into national immunization programmes to mitigate morbidity and mortality associated rotavirus diarrhoea. Lower vaccine effectiveness has however been noted in low-middle income countries, and much research has been done to understand factors associated with this; among them are components found in breast milk. This study assessed the impact of lactoferrin, lactadherin, and tenascin-c on rotavirus vaccine seroconversion.

## Materials and methods

From a cohort of 420 mother-infant pairs, 128 were included in the study. Serum samples were collected from the infant at baseline and one month after second rotavirus vaccine dose. Breast milk samples were collected from mothers at baseline. Determination of titres of rotavirus-specific immunologlobulin G and A in breast milk and serum as well as concentrations of lactoferrin, lactadherin, and tenascin-c were done using standard ELISA. Antibody and concentration variables were modelled on log-base 2 and multivariate analysis was applied to adjust for all breast milk factors in the final model.

## Results

We found evidence of an association between exposure to high lactadherin concentration in breast milk and failure to seroconvert by the infant; a two-fold increase in breast milk lactadherin increased by 12% the risk of not seroconverting after rotavirus vaccination (Crude RR**=**1.12; 95% CI=1.03-1.22; P=<0.001). This association remained after adjusting for covariates, decreasing slightly to 10% (Adjusted RR=1.10; 95% CI=1.01- 1.20; P=0.002).

## Conclusion

High breast milk concentrations of lactadherin may be a significant contributor to poor performance of rotavirus vaccines. More research is recommended to further elucidate this observation.

# Introduction

Poor efficacy of orally administered rotavirus vaccines in many low and middle income countries (LMICs) has spurred great efforts towards understanding the reasons behind this observed trend [1–4]. Several factors explaining this reduced efficacy have been postulated including malnutrition, concurrent administration with other vaccines, infection with various enteric pathogens, environmental enteropathy, host genetic and maternal factors [5–14].

Maternal factors affecting rotavirus vaccine efficacy have become a focus of attention with research showing that in addition to immunologic components of breast milk (immunoglobulin A & B, (IgA and IgG)), non-immunologic components may also play a role in reduced vaccine efficacy [15]. Moon and colleagues (2013) showed that women from LMICs had higher rotavirus specific antibodies as well as higher levels of non-antibody breast milk antiviral glycoproteins, including lactoferrin (LF) and lactoadherin (LA), and demonstrated that these non-antibody components contributed to increased neutralization activity of breast milk against rotavirus [15]. Tenascin-C (TNC) has also recently become the focus of research as another non-immunological breast milk component that has shown activity against the HIV virus and might therefore contribute to breast milk viral neutralizing properties [16].

Lactoferrin is a highly multi-functional protein found in secretory fluids such as tears, saliva, nasal secretions and breast milk [17]. It has been well researched for its anti-bacterial, anti-fungal and anti-viral properties [18–20]. Lactadherin is a milk fat protein also noted for its antiviral properties [21], while TNC is a highly conserved extracellular glycoprotein that has been previously associated with fetal growth development and wound healing properties [22,23]. Recent work has shown that TNC protein may inhibit HIV-1 infectivity through blocking of the HIV-1 envelope interaction with mucosal epithelial cells [16].

Based on this theoretical evidence, we hypothesized that the presence of these three proteins in breast milk could have an impact on the ability of an infant to mount an adequate immune response to oral live virus vaccination. In the gut, rotavirus interacts with mucosal epithelial cells receptors through which it is engulfed and subsequently uncoated out of the virion in order to replicate during an infection [24]. The live attenuated rotavirus vaccine aims to replicate similarly in order release transcriptionally active particles that trigger an immunized child to mount an immune response. Our hypothesis is that during rotavirus vaccination, the anti-viral activities of LF, LA and TNC present in breast milk interacts with the vaccine, inhibiting the live virus from entering the mucosal cells.

This study investigated the effect of maternal breast milk levels of TNC, LA and LF in Zambian women on their infant’s seroconversion to the monovalent vaccine Rotarix™ (GlaxoSmithKline Biologicals, Rixensart, Belgium) when routinely immunized within the expanded programme on immunization in Zambia.

# Materials and methods

## Study site and participants

The study site and participant enrolment was as described previously [25]. Briefly, a population of eligible and consenting mothers and their infants attending routine immunization at Kamwala Clinic in Lusaka Zambia were recruited into the study. At 6 weeks post-partum (baseline), a sample of breast milk was provided by the mother through manual expression and immediately stored at -80^o^C. Blood was drawn from their infants at the time of breast milk collection for measurement of rotavirus-specific IgA and IgG described in detail previously [25] after which the infants received the routinely scheduled immunizations including rotavirus vaccine, Rotarix™. A second blood draw was taken from infants one month after receiving the second dose of Rotarix™ to measure rotavirus-specific IgA which was used to determine the seroconversion status of the infants.

The study protocol was approved by the University of Zambia Biomedical Research Ethics Committee, University of North Carolina at Chapel Hill Institutional Review Board and the Zambian Ministry of Health. The study was conducted in accordance with the principles of the Declaration of Helsinki and in compliance with good clinical practice guidelines; ClinicalTrials.gov registration number NCT 01886833.

## Measurement of LF, LA and TNC in breast milk

Enzyme-linked immunosorbent assay (ELISA) was used for measurement of LF, LA and TNC in breast milk samples. LF concentrations were determined using commercial Human Lactoferrin (HLF2) ELISA kit (ab108882 Abcam, Cambridge United Kingdom) according to the manufacturer’s instructions except that the aqueous portion of breast milk samples was serially diluted to 1:1,000,000. Briefly, breast milk samples were centrifuged at 800 x *g* for 10 minutes and the diluted aqueous portion added to microplate wells pre-coated with LF antibody and incubated. After washing, wells were treated with human LF-specific biotinylated detection antibody, followed by another wash step and incubated with streptavidin peroxidase conjugate. After incubation, color reactions were developed with chromogen substrate, 3,3’,5,5’-tetramethylbenzidine, and stopped with stop solution. Optical density was read at 450nm and 570nm for wavelength correction and LF concentrations were determined using assay standard curve. Commercial ELISA kits were similarly used to determine concentrations of LA (LS-F12031, LifeSpan BioSciences USA) and TNC (LS-F22079, LifeSpan BioSciences USA). Samples were centrifuged at 800 x g for 10 minutes, LA samples were left undiluted and TNC diluted 1:100 and assayed as with LF substituting Avidin-Horseradish Peroxidase during color development. Optical density was read at 450nm wavelength and concentrations were determined based on the assay standard curve.

## Sample size consideration

## We calculated the post-hoc power for a given sample size of 128 available for this study. For a 40% prevalence of non-seroconverted infants one month following full rotavirus immunisation, this study is powered at about 70% to detect a decrease to 20% in the proportion of babies not seroconverted using a two-sided Pearson’s chi-squared test at 5% level of significance.

## Statistical analysis

Vaccine seroconversion in infants was defined as a four-fold or greater increase in rotavirus-specific IgA titre between baseline and 1 month post Rotarix™ second dose. IgA titre higher or equal to 40 in serum was considered positive. IgA titre smaller than 20 (below limit of detection) in serum was assigned a value of 1. We summarised breast milk components by key infant and maternal factors using proportions. Pearson Chi-squared test was used to determine the relationships between failed vaccine seroconversion and breast milk glycoproteins (TNC, LA and LF), maternal and child characteristics. For breast milk rotavirus specific-IgA and infant serum rotavirus specific-IgG categorized as quartiles, Wilcoxon rank-sum test for trend was used. Relationships between the breast milk antiviral glycoproteins (on continuous scale) were determined using Spearman’s correlation coefficient. Where there was no evidence of pairwise correlation, we included all the components in the regression model to assess independent effects.

Generalised linear model with log-link binomial-family was used to estimate the independent effects of non-immunological factors on probability of non-seroconversion adjusting for infant and maternal factors. The non-immunological factors were transformed into log base 2 scales and modelled as a continuous covariate so that the effect would be interpreted as a doubling of antibody response. In the multivariable model, we estimated the independent as well as joint effects of the non-immunologic factors on non-seroconversion. Covariates (i.e. breast milk, maternal and child characteristics) were retained in the model regardless of their p-values to improve the precision of the estimates. Correlation between variables was explored and possible interactions between variables explored within the model. Data were analysed using Stata 14 (StataCorp, College Station, Texas, USA).

# Results

A total of 420 mother-infant pairs successfully enrolled in the primary study. Information on non-immunological components was collected from 262 and information on seroconversion from 134. This was partly due to participants’ failure to bring infants for post vaccination blood draws and drop out after initial enrolment. The final sample for analysis comprised a total of 128 individuals, as shown in Fig 1.

Fig 1. Study participant’s flow chart

Presence of non-immunological components was found in 260 out of 262 samples (99%) for TNC and 251 out of 262 for LF (96%). For LA, the biomarker was found in 116 out 259 samples (45% of the total) as shown in Table 1.

Stratified by seroconversion status, LA was found in 61% of the breast milk samples from mothers with babies who failed to serconvert and 36% of those with seroconverted babies. Presence of TNC and LF was found in over 95% of samples regardless of the seroconversion status of their babies.

| Table 1. Prevalence of TNC, LA and LF by key infant and maternal characteristics | | | | | |
| --- | --- | --- | --- | --- | --- |
| Characteristics | Number of Infants | TNC | LA | LF | Any biomarker |
| Sex of child |  |  |  |  |  |
| Female | 152 | 151 (99) | 71 (47) | 145 (95) | 152 (100) |
| Male | 110 | 109 (99) | 45 (41) | 106 (96) | 110 (100) |
| Age of child at vaccination (Weeks) |  |  |  |  |  |
| <7 | 161 | 160 (99) | 68 (42) | 152 (94) | 161 (100) |
| 7+ | 101 | 100 (99) | 48 (48) | 99 (98) | 101 (100) |
| Seroconversion after second vaccine dose |  |  |  |  |  |
| No | 49 | 48 (98) | 30 (61) | 47 (96) | 49 (100) |
| Yes | 85 | 85 (100) | 31 (36) | 82 (96) | 85 (100) |
| Seropositivity at baseline (IgA>1:40) |  |  |  |  |  |
| No | 171 | 169 (99) | 75 (44) | 165 (96) | 171 (100) |
| Yes | 58 | 58 (100) | 25 (43) | 56 (97) | 58 (100) |
| Maternal age |  |  |  |  |  |
| 16-19 | 36 | 36 (100) | 16 (44) | 35 (97) | 36 (100) |
| 20-29 | 168 | 166 (99) | 74 (44) | 160 (95) | 168 (100) |
| 30-39 | 58 | 58 (100) | 26 (45) | 56 (97) | 57 (98) |
| Maternal HIV Status |  |  |  |  |  |
| Negative | 183 | 182 (99) | 78 (43) | 179 (98) | 183 (100) |
| Positive | 77 | 76 (99) | 37 (48) | 71 (92) | 77 (100) |
| Breast milk anti-rotavirus IgA - Quartiles (median titre) | | |  |  |  |
| 1 (40) | 52 | 51 (98) | 23 (44) | 51 (98) | 52 (100) |
| 2 (80) | 53 | 53 (100) | 21 (40) | 51 (96) | 53 (100) |
| 3 (160) | 49 | 49 (100) | 25 (51) | 46 (94) | 49 (100) |
| 4 (640) | 82 | 82 (100) | 41 (50) | 80 (98) | 82 (100) |
| Breast milk anti-rotavirus IgG - Quartiles (median titre) | | |  |  |  |
| 1 (160) | 46 | 45 (98) | 16 (35) | 43 (93) | 46 (100) |
| 2 (2560) | 33 | 33 (100) | 11 (33) | 32 (97) | 33 (100) |
| 3 (5120) | 51 | 51 (100) | 23 (45) | 49 (96) | 51 (100) |
| 4 ( 20480) | 100 | 99 (99) | 50 (50) | 98 (98) | 100 (100) |
| Season of vaccination |  |  |  |  |  |
| Dry (May-October) | 148 | 147 (99) | 62 (42) | 140 (95) | 148 (100) |
| Wet (November-April) | 112 | 111 (99) | 53 (47) | 109 (97) | 112 (100) |
| Total | 262 | 260 (99) | 116 (45) | 251 (96) | 262 (100) |

Overall, 37% (49/134) of infants did not seroconvert following rotavirus vaccination. Infants whose mothers had detectable LA (*P*<0.01) and higher titres of rotavirus-specific IgA in breast milk ( *P*<0.01) were less likely to seroconvert. Similarly, those with HIV-positive mothers were also less likely to seroconvert (*P*=0.04) as shown in Table 2.

| Table 2. Frequency of seroconversion after second vaccine dose by breast milk components and key infant and maternal factors among rotavirus-vaccinated infants aged 6 - 11 weeks. | | | | |
| --- | --- | --- | --- | --- |
|  |  |  |  |  |
| Biomarker | Number of Infants (% of total) | No. (%) not seroconverted | 95% CI | Chi2 *P*-value |
| **TNC** |  |  |  |  |
| No | 1 (1) | 1 (100) | -* | 0.19 |
| Yes | 133 (99) | 48 (36) | (28.3-44.7) |  |
| **LA** |  |  |  |  |
| No | 71 (53) | 18 (25) | (16.5-36.9) | **<0.01** |
| Yes | 61 (46) | 30 (49) | (36.7-61.7) |  |
| **LF** |  |  |  |  |
| No | 5 (4) | 2 (40) | (8.1-83.4) | 0.87 |
| Yes | 129 (96) | 47 (36) | (28.5-45.2) |  |
| **Any** |  |  |  |  |
| No | 0 (-) | - | -* | - |
| Yes | 134 (100) | 49 (37) | (28.8-45.1) |  |
| **Sex of child** | |  |  |  |
| Female | 74 (55) | 30 (41) | (29.8-52.2) | 0.29 |
| Male | 60 (45) | 19 (32) | (21.0-44.6) |  |
| **Age of child at vaccination (Weeks)** | | |  |  |
| <7 | 82 (61) | 27 (33) | (23.5-43.9) | 0.27 |
| 7+ | 52 (39) | 22 (42) | (29.5-56.2) |  |
| **Seropositivity at baseline (IgA>1:40)** | |  |  |  |
| No | 102 (76) | 35 (34) | (25.7-44.2) | 0.27 |
| Yes | 31 (23) | 14 (45) | (28.5-63.0) |  |
| **Maternal age** | |  |  |  |
| 16-19 | 22 (16) | 9 (41) | (22.4-62.5) | 0.43 |
| 20-29 | 85 (63) | 33 (39) | (29.0-49.7) |  |
| 30-39 | 27 (20) | 7 (26) | (12.6-45.9) |  |
| **Maternal HIV Status** | |  |  |  |
| Negative | 89 (66) | 27 (30) | (21.6-40.8) | **0.04** |
| Positive | 45 (34) | 22 (49) | (34.5-63.5) |  |
| **Breast milk anti-rotavirus IgA - Quartiles (median titre)** | | | |  |
| 1 (1-40) | 32 (24) | 7 (22) | (10.6-39.8) | **<0.01** |
| 2 (80) | 29 (22) | 10 (34) | (19.3-53.6) |  |
| 3 (160) | 25 (19) | 8 (32) | (16.5-52.8) |  |
| 4 (640) | 45 (34) | 24 (53) | (38.6-67.5) |  |
| **Breast milk anti-rotavirus IgG - Quartiles (median titre)** | | | |  |
| 1 (160) | 31 (23) | 7 (23) | (10.9-40.9) | 0.06 |
| 2 (2560) | 17 (13) | 5 (29) | (12.3-55.2) |  |
| 3 (5120) | 26 (19) | 11 (42) | (24.8-62.0) |  |
| 4 ((10,240)) | 59 (44) | 25 (42) | (30.3-55.4) |  |
| **Season of vaccination** | |  |  |  |
| Dry (May-October) | 80 (60) | 27 (34) | (24.1-44.9) | 0.41 |
| Wet (November-April) | 54 (40) | 22 (41) | (28.3-54.4) |  |
| **Total** | 134 | 49 (37) | (29-45) |  |
| *No infant seroconverted in this category, therefore it was not possible to calculate a confidence interval for it. | | | | |
|  |  |  |  |  |

**Factors independently associated with lactadherin**

In our non-adjusted analysis, we found evidence of a correlation between LA and failure to seroconvert; a two-fold increase in breast milk LA would result in a 12% increase in the risk of not seroconverting to the vaccine (Crude RR**=**1.12; 95% CI=1.03-1.22; *P*=<0.01) Table 3. This association remained stable after adjusting for key covariates (Adjusted RR=1.10; 95% CI=1.01- 1.20; *P*<0.012). Neither the individual effects of TNC (Adjusted RR=1.0; 95% CI=0.78- 1.31; *P*=0.95) and LF (Adjusted RR=0.99; 95% CI=0.93- 1.06); *P*=0.84) nor the joint effect of the three non-immunologic factors (Adjusted RR=1.37; 95% CI=0.79- 2.35; *P*=0.26) were found to be correlated with the risk of not seroconverting in the crude and adjusted models as summarised in Table 3. We didn’t find any correlations between the study covariates. Similarly, no interactions between covariates were statistically significant in any of our models.

| Table 3. Effects of non-immunological factors in breast milk on risk ratio of not seroconverting post second dose among rotavirus-vaccinated infants aged 6 - 11 weeks. | | | | |
| --- | --- | --- | --- | --- |
| Non-immunologic Factor | Crude RR (95%CI) | *P*-value | Adjusted RR (95%CI) ^1^ | Adjusted P-value |
| **TNC** |  | |  |  |
| No | Reference | 0.59 | reference | 0.95 |
| Yes | 0.93 (0.70, 1.22) |  | 1.00 (0.78, 1.31) |  |
| **LA** |  |  |  |  |
| No | Reference | **<0.01** | reference | **<0.01** |
| Yes | 1.12 (1.03, 1.22) |  | 1.10 (1.01, 1.20) |  |
| **LF** |  |  |  |  |
| No | Reference | 0.68 | reference | 0.82 |
| Yes | 0.99 (0.94, 1.04) |  | 0.99 (0.93, 1.06) |  |
| **Joint effect TNC, LA, LF^2^** | |  |  |  |
| No | Reference | 0.96 | reference | 0.55 |
| Yes | 1.01 (0.64, 1.59) |  | 1.16 (0.7, 1.96) |  |
| ^1^ Estimates were adjusted for Breast milk anti-rotavirus IgA (transformed on log base 2); Infant serum anti-rotavirus IgG titre (transformed on log base 2); Maternal age (categorical); Maternal HIV status; Seropositivity at baseline (IgA >= 1:40) (binary); Age of child at vaccination (binary); Sex and TNC, LA and LF (transformed on log base 2) | | | | |
| ^2^Joint effect was calculated using linear combination of TNC, LA and LF and estimated using the lincom command. | | | | |

# Discussion

Analysis of our results showed a 12% increase in risk of failure to seroconvert for every two-fold increase in breast milk LA, and that neither TNC nor LF nor the joint effect of all 3 non-immunologic factors had any effect on seroconversion.

Our results differ slightly from the results reported by Moon and colleagues [15] who indicated an increase in virus neutralization activity with increased LF and LA concentrations. One key consideration is that their results were observed in commercial LF and LA preparations [15], which would not take into account any interactions that may be taking place within the natural breast milk sample as we have assessed. This disparity highlights the need to *in-vitro* assays to be validated *in-vivo*, to ensure that reported findings have real-life validity.

Our results showed mean concentrations of LF to be 50.3mg/l. These results are closer to those reported in Australia [26] and Democratic Republic of Congo [27], but are significantly lower than those reported in Malawi [28] , Gambia [29], Bangladesh [30] and Nigeria [31]. The differences in observed results for LF may be explained by methodological differences in analysis of samples, and the time post-partum, at which the the breast milk samples were collected; it is well known that the moieties in such fluids as breast milk change with time. A number of these studies were conducted when ELISAs were not commonly used and as such, they made use of methods such as radioimmunoassay and chromatographic techniques which are not presently used. Data from Malawi was obtained using an ELISA, whilst that of Houghton and colleagues [26] made use of antibodies harvested from animals within their own lab in EIAs. Further, the established variation in breast milk samples from the aqueous and lipid portion separation which many studies encounter but do not adjust for, may be important when interpreting these finding as alluded to by Filteau and collegues [30].

Another possible explanation for the observed differences in concentration is the known affinity of LF for other biological molecules. LF has been found to bind to IgA, casein, albumin, lysozymes and lactoglobulins [32] and could be rendered unavailable for binding during testing. This calls for further analysis of samples to possibly exclude these interactions before testing for effect of individual components such as LF. However, it could still be argued that the amount of free LF is best measured in the presence of such interactions.

Very little is known on LA concentrations in breast milk with only one study from Mexico [21] which reported much higher concentrations than those observed in our cohort (mean concentration 96.53 pg/ml). The higher concentration measures could be attributed to the use of end point recombinant immunoblot assay (RIBA) as opposed to standard ELISA. Newburg and colleagues (1998) noted that higher LA concentrations were able to play a key role in mitigating rotavirus symptomatic infection even in the absence of LF [21]. This observation would best account for the increase in risk of 12% attributable to LA observed during our analysis.

Similar to LA, TNC research has been limited with regards to determination of its breast milk concentrations in various cohorts, but recent work found concentrations much lower than those reported here (mean concentration 1.10mg/ml) [33]; although the main study tested TNCs neutralizing ability with regards to HIV-1. Similar work from Nicaragua failed to find any association between seroconversion and innate immune factors including TNC, however the small sample size may have been insufficient to detect significant effects [34]. Nonetheless, we noted a similar result from our analysis suggesting that breast milk TNC may have no effect on live virus uptake in the gut .

Although our results showed that the presence of TNC, LA, and LF together did not have an effect on rotavirus vaccine seroconversion among the infants, further work is needed before a conclusion can be made. An analysis of this nature may clarify the work by Moon et al [15] who noted that as concentrations of LF and LA increased so did rotavirus neutralization. Additionally, nearly all mothers had detectable TNC and LF levels in breast milk; this could be a limiting factor in analysis of the individual effect of LA as it could have been masked behind the total effect of all three non-immunological factors. Thus, we recommend further research on the effect of LA and its ability to affect rotavirus seroconversion in infants in the absence of the other two factors may be of interest.

Our analysis also showed that fewer children of HIV positive mothers seroconverted than those of uninfected mothers (*P*=0.035) which did not remain significant after adjusting for LA, a result previously unobserved in our cohort (24) or indeed lack of differences in the vaccine effectiveness study findings [35]. Our previous work (24) focused on the children that had seroconversion data at the time of analysis however, in this study sample selection took into account those who had known seroconversion status as well as data on all three biological markers LA LF and TNC; therefore, presented here is slightly different and smaller sample.

The result of HIV exposure being significant is of interest to us for various reasons. Despite literature showing that infants are able to mount adequate immune responses to rotavirus vaccine regardless of their HIV exposure status [36–38], it once again reminds us that HIV exposed children present a challenging population subset that require careful understanding of how the dynamics of exposure may impact their outcomes in the various areas of research. Indeed recent meta-analysis findings by Brenan et al (2016) showed that there is a consistently observed increased risk of all-cause mortality for HIV exposed uninfected infants compared to HIV unexposed infants [39]. Our data here suggests that the presence of LA could have some association with HIV exposure status of children. This however is another area for further research with regards to the mode of actions of these factors on rotavirus vaccines in the context of HIV.

# Conclusions

We conclude that while our study was not able to show any statistically significant joint effect of breast milk glycoprotein LA, LF and TNC on rotavirus vaccine seroconversion in infants, we did however demonstrate a 12% increase in risk of failure to seroconvert in infants with a 2-fold increase in LA. There is need for further work to determine if breast milk concentrations LA and rotavirus IgA contribute to the lower seroconversion rate in infants after rotavirus vaccination in developing regions.

# Acknowledgements

We are grateful to the mothers and infants enrolled in the study cohort. We also thank the study team including Marcellina Hamikondo, Fridah Madhabi, Margaret Chisambi, Annie Sinyangwe, Catherin Phiri and Annie Chikombo for directly working on this study.

The primary study is funded by the National Institutes for Health (NIH), USA, through an R01 grant 1R01AI099601. Non-immunological components of breast milk study was funded by the Zambia National Science and Technology Council NSTC/101/5/10.

# Author Contributions

Conceived project: RC, MS

Conducted research and analysis: KMK, MS, NL, CCC

Data analysis: MPG, SB

Manuscript Development and Revision KMK, MS, NL, CCC, MPG, SM, SP, SB, MPG, RC

# References

1. Vesikari T, Karvonen A, Puustinen L, Zeng S-Q, Szakal ED, Delem A, et al. Efficacy of RIX4414 Live attenuated human rotavirus vaccine in Finnish infants. Pediatr Infect Dis J. 2004;23.

2. Zaman K, Dang DA, Victor JC, Shin S, Yunus M, Dallas MJ, et al. Efficacy of pentavalent rotavirus vaccine against severe rotavirus gastroenteritis in infants in developing countries in Asia: a randomised, double-blind, placebo-controlled trial. Lancet (London, England). England; 2010;376: 615–623. doi:10.1016/S0140-6736(10)60755-6

3. Madhi SA, Cunliffe NA, Steele D, Witte D, Kirsten M, Louw C, et al. Effect of Human Rotavirus Vaccine on Severe Diarrhea in African Infants. N Engl J Med. 2010;362: 289–298. doi:10.1056/NEJMoa0810625)

4. Ruiz-Palacios GM, Pérez-Schael I, Velázquez FR, Abate H, Breuer T, Clemens SC, et al. Safety and efficacy of an attenuated vaccine against severe rotavirus gastroenteritis. N Engl J Med. Massachusetts Medical Society; 2006;354: 11–22. doi:10.1056/NEJMoa052434

5. Moon S, Yuhuan WM, Shane AL, Trang N, Ray P, Dennehy P, et al. Inhibitory effect of breast milk in infectivity of live oral rotavirus vaccines. Pediatr Infect Dis J. 2010;29: 919–923. doi:10.1097/INF.0b013e3181e232ea.Inhibitory

6. Moon SS, Groome MJ, Velasquez DE, Parashar UD, Jones S, Koen A, et al. Prevaccination Rotavirus Serum IgG and IgA Are Associated with Lower Immunogenicity of Live, Oral Human Rotavirus Vaccine in South African Infants. Clin Infect Dis. 2016;62: 157–165. doi:10.1093/cid/civ828

7. Lopman BA, Pitzer VE, Sarkar R, Gladstone B, Patel M, Glasser J, et al. Understanding reduced rotavirus vaccine efficacy in low socio-economic settings. PLoS One. 2012;7. doi:10.1371/journal.pone.0041720

8. Gilmartin AA, Petri WAJ. Exploring the role of environmental enteropathy in malnutrition, infant development and oral vaccine response. Philos Trans R Soc Lond B Biol Sci. 2015;370. doi:10.1098/rstb.2014.0143

9. Petri WA, Miller M, Binder HJ, Levine MM, Dillingham R, Guerrant RL. Enteric infections, diarrhea, and their impact on function and development. J Clin Invest. 2008;118: 1277–1290. doi:10.1172/JCI34005

10. Prendergast AJ. Malnutrition and vaccination in developing countries. Philos Trans R Soc B Biol Sci. 2015;370: 20140141–20140141. doi:10.1098/rstb.2014.0141

11. Naylor C, Lu M, Haque R, Mondal D, Buonomo E, Nayak U, et al. EBioMedicine Environmental Enteropathy , Oral Vaccine Failure and Growth Faltering in Infants in Bangladesh. EBIOM. The Authors; 2015;2: 1759–1766. doi:10.1016/j.ebiom.2015.09.036

12. Nordgren J, Sharma S, Bucardo F, Nasir W, Günaydin G, Ouermi D, et al. Both lewis and secretor status mediate susceptibility to rotavirus infections in a rotavirus genotype-dependent manner. Clin Infect Dis. 2014;59: 1567–1573. doi:10.1093/cid/ciu633

13. Taniuchi M, Platts-Mills JA, Begum S, Uddin MJ, Sobuz SU, Liu J, et al. Impact of enterovirus and other enteric pathogens on oral polio and rotavirus vaccine performance in Bangladeshi infants. Vaccine. Netherlands; 2016;34: 3068–3075. doi:10.1016/j.vaccine.2016.04.080

14. Nakaya HI, Bruna-romero O. Is the gut microbiome key to modulating vaccine ? Expert Rev Vaccines. 2015;14: 777–779. doi:10.1586/14760584.2015.1040395

15. Moon S, Ray P, Dennehy P, Glass RI. Differential profiles and inhibitory effect on rotavirus vaccines of nonantibody components in breast milk from mothers in developing and developed countries. Pediatr Infect Dis J. 2013;32: 863–870. doi:10.1097/INF.0b013e318290646d

16. Fouda GG, Jaeger FH, Amos JD, Ho C, Kunz EL, Anasti K, et al. Tenascin C is an innate broad spectrum HIV-1 – neutralizing protein in breast milk. PNAS. 2013;110: 18220–18225. doi:10.1073/pnas.1307336110/-/DCSupplemental.www.pnas.org/cgi/doi/10.1073/pnas.1307336110

17. Sánchez L, Calvo M, Brock JH. Biological role of lactoferrin. Arch Dis Child. 1992;67: 657–661. doi:10.1136/adc.67.5.657

18. Harmsen MC, Swart PJ, Béthune M-P de, Pauwels R, Clercq E De, The TB, et al. Antiviral Effects of Plasma and Milk Proteins: Lactoferrin Shows Potent Activity against Both Human Immunodeficiency Virus and Human Cytomegalovirus Replication In Vitro. J Infect Dis. 1995;172: 380–388.

19. Puddu P, Borghi P, Gessani S, Valenti P, Belardelli F, Seganti L. Antiviral effect of bovine lactoferrin saturated with metal ions on early steps of human immunodeficiency virus type 1 infection. Int J Biochem Cell Biol. Netherlands; 1998;30: 1055–1062.

20. Nozaki A, Ikeda M, Naganuma A, Nakamura T, Inudoh M, Tanaka K, et al. Identification of a Lactoferrin-derived Peptide Possessing Binding Activity to Hepatitis C Virus E2 Envelope Protein. J Biol Chem . 2003;278: 10162–10173. doi:10.1074/jbc.M207879200

21. Newburg DS, Peterson JA, Ruiz-Palacios GM, Matson DO, Morrow AL, Shults J, et al. Role of human-milk lactadherin in protection against symptomatic rotavirus infection. Lancet. 1998;351: 1160–1164. doi:10.1016/S0140-6736(97)10322-1

22. Midwood KS, Orend G. The role of tenascin-C in tissue injury and tumorigenesis. J Cell Commun Signal. Dordrecht: Springer Netherlands; 2009;3: 287–310. doi:10.1007/s12079-009-0075-1

23. Imanaka-Yoshida K, Aoki H. Tenascin-C and mechanotransduction in the development and diseases of cardiovascular system. Front Physiol. Frontiers Media S.A.; 2014;5: 283. doi:10.3389/fphys.2014.00283

24. Isa P, Gutiérrez M, Arias CF, López S. Rotavirus cell entry. Future Virol. Future Medicine; 2008;3: 135–146. doi:10.2217/17460794.3.2.135

25. Roma Chilengi1, Michelo Simuyandi, Lauren Beach, Katayi Mwila SB-, Dreps, Devy M. Emperador, Daniel E. Velasquez, Samuel Bosomprah BJ. Association of Maternal Immunity with Rotavirus Vaccine Immunogenicity in Zambian Infants. PLoS One. 2016;

26. Houghton MR, Gracey M, Burke V, Bottrell C, Spargo RM. Breast Milk Lactoferrin Levels in Relation to Maternal Nutritional Status. J Pediatr Gastroenterol Nutr. 1985;4.

27. Hennart PF, Brasseur DJ, Delogne-Desnoeck JB, Dramaix MM, Robyn CE. Lysozyme, lactoferrin, and secretory immunoglobulin A content in breast milk: Influence of duration of lactation, nutrition status, prolactin status, and parity of mother. Am J Clin Nutr. 1991;53: 32–39.

28. Semba RD, Kumwenda N, Taha TE, Hoover DR, Lan Y, Eisinger W, et al. Mastitis and Immunological Factors in Breast Milk of Lactating Women in Malawi. Clin Diagn Lab Immunol. American Society for Microbiology; 1999;6: 671–674.

29. Prentice A, Prentice AM, Lamb WH. Mastitis in rural Gambian mothers and the protection of the breast by milk antimicrobial factors. Trans R Soc Trop Med Hyg. England; 1985;79: 90–95.

30. Filteau SM, Rice AL, Ball JJ, Chakraborty J, Stoltzfus R, de Francisco A, et al. Breast milk immune factors in Bangladeshi women supplemented postpartum with retinol or {beta}-carotene. Am J Clin Nutr. 1999;69: 953–958.

31. Ella EE, Ahmad AA, Umoh VJ, Ogala WN, B BT. Lactoferrin levels in human breast milk among lactating mothers with sick and healthy babies in Kaduna State, Nigeria. International Journal of Medicine and Medical Sciences. Academic Journals; 2009. pp. 495–500.

32. Lampreave F, Pineiro A, Brock JH, Castillo H, Sanchez L, Calvo M. Interaction of bovine lactoferrin with other proteins of milk whey. Int J Biol Macromol. Netherlands; 1990;12: 2–5.

33. Mansour RG, Stamper L, Jaeger F, McGuire E, Fouda G, Amos J, et al. The Presence and Anti-HIV-1 Function of Tenascin C in Breast Milk and Genital Fluids. PLoS One. Public Library of Science; 2016;11: e0155261. doi:10.1371/journal.pone.0155261

34. Becker-dreps S, Choi WS, Stamper L, Vilchez S, Velasquez DE, Moon S. Innate immune factors in mothers â€^TM^ breast milk and their lack of association with rotavirus vaccine immunogenicity in Nicaraguan infants. J Pediatr Infect Dis Soc. 2015; 1–4. doi:10.1093/jpids/piv076

35. Beres LK, Tate JE, Njobvu L, Chibwe B, Rudd C, Guffey MB, et al. A Preliminary Assessment of Rotavirus Vaccine Effectiveness in Zambia. Clin Infect Dis. 2016;62: S175–S182. doi:10.1093/cid/civ1206

36. Groome MJ, Madhi S a. Five-year cohort study on the burden of hospitalisation for acute diarrhoeal disease in African HIV-infected and HIV-uninfected children: potential benefits of rotavirus vaccine. Vaccine. 2012;30 Suppl 1: A173-8. doi:10.1016/j.vaccine.2011.08.004

37. Steele AD, Madhi SA, Louw CE, Bos P, Tumbo JM, Werner CM, et al. Safety, reactogenicity, and immunogenicity of human rotavirus vaccine RIX4414 in human immunodeficiency virus-positive infants in South Africa. Pediatr Infect Dis J. 2011;30.

38. Obaro SK, Pugatch D, Luzuriaga K. Immunogenicity and efficacy of childhood vaccines in HIV-1-infected children. Lancet Infect Dis. 2004;4: 510–8. doi:10.1016/S1473-3099(04)01106-5

39. Brennan AT, Bonawitz R, Gill CJ, Thea DM, Kleinman M, Useem J, et al. A meta-analysis assessing all-cause mortality in HIV-exposed uninfected compared to HIV-unexposed uninfected infants and children. AIDS. 2016;30: 2351–2360.
